# The Ce–Ni–Si System Revisited: More Homologue Compounds?

**DOI:** 10.1021/acs.inorgchem.3c04594

**Published:** 2024-05-02

**Authors:** Fainan Failamani, Andriy Grytsiv, Jiri Bursik, Gerald Giester, Peter Rogl

**Affiliations:** † Institute of Materials Chemistry, University of Vienna, Währingerstraße 42, Vienna A-1090, Austria; ‡ Division of Inorganic and Physical Chemistry, Faculty of Mathematics and Natural Sciences, Institut Teknologi Bandung, Jalan Ganesha 10, Bandung 40132, Indonesia; § Institute of Physics of Materials, Czech Academy of Sciences, Žižkova 22, Brno 61662, Czech Republic; ∥ Institute of Mineralogy and Crystallography, University of Vienna, Josef-Holaubek-Platz 2, Vienna A-1090, Austria

## Abstract

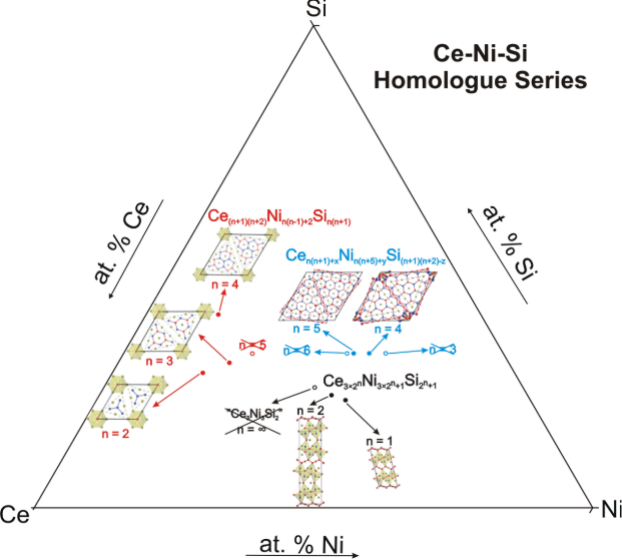

The nickel-rich region of the system Ce–Ni–Si has been reinvestigated utilizing X-ray single-crystal, powder, and electron diffraction as well as electron microprobe and thermal analyses. Two novel hexagonal compounds, τ-Ce_20+*x*_Ni_36+*y*_Si_30–*z*_ and τ′-Ce_30+*x*_Ni_50+*y*_Si_42–_*_z_,* were identified. The crystal structure of τ-Ce_20+*x*_Ni_36+*y*_Si_30–*z*_ was derived from single-crystal X-ray diffraction and found to be isotypic with the Sm_10_Ni_20.8_P_15_-type structure (S.G. *P*6_3_/*m,x* = 1.8, *y* = 3.0, *z* = 1.8, *a* = 2.07156(2) nm, *c* = 0.39990(1) nm, *R*_F_ = 0.048). Rietveld refinement of τ′-Ce_30+*x*_Ni_50+*y*_Si_42–*z*_ revealed isotypism with Tb_15_Ni_28_P_21_ (S.G. *P*6_3_/*m*, *a* = 2.46926(13) nm, *c* = 0.40019(3) nm, *R*_F_ = 0.058). The compound Ce_3_Ni_4_Si_2_ from X-ray single-crystal analysis was found to crystallize in a novel structure type with monoclinic unit cells (S.G. *C*2/*c*, *a* = 1.54708(3) nm, *b* = 0.58677(1) nm, *c* = 0.74331(1) nm, β = 102.985(1)°, *R*_F_ = 0.017). This compound belongs to a new homologue series in the RE–Ni-Si system (RE = La and Ce) with general formula of RE_(3×2^*n*^)_Ni_(3×2^*n*^ + 1)_Si_(2^*n*+1^)_; *n* = 0,1, ..., ∞. The crystal structure of this series is characterized by alternating numbers (2^*n*^) of corner-sharing Si-polyhedral blocks sandwiched between zigzag nickel chains. Higher-order members of this series are produced by the formation of more corner-sharing Si-polyhedral blocks due to removal of nickel chains.

## Abstract 2

Two homologue series were discovered in the Ce−Ni−Si system, with the possibility of discovery of new members and/or series.

## Introduction

Ternary systems containing rare earth (RE), transition metals (TM), and silicon are hosts of many intermetallic compounds with various crystal structure types and physical properties. Systems containing lanthanum and cerium are widely studied since those metals are the most abundant REs in earth’s crust. Moreover, competing interactions on the Ce 4f^1^ electron gave rise to various interesting phenomena such as valence instabilities, heavy Fermion behavior, magnetic ordering, unconventional superconductivity, thermoelectricity.^1,2^

Studies of the ternary Ce-TM-Si systems were started over four decades ago,^3−6^ with an exception for the Zn-containing system, which was reported more recently.^7,8^ Among ternary Ce-TM-Si systems, the system with nickel exhibits the largest number of ternary phases and structure types.^9^ Since the first study in 1969, more ternary Ce–Ni–Si compounds have been discovered;^10−13^ however, for some compounds, their crystal structures still remain unresolved, *e.g*., CeNi_1.3_Si_0.7_ and Ce_27_Ni_43_Si_30_.^12^

During our latest work on the BaAl_4_ derivative phases in the {La,Ce}-(Ni,Zn)-Si systems, we discovered a new phase labeled as Ce(Ni_1–*x*_Si_*x*_)_3_.^10^ Further investigation into the new phase led us to discover another phase with close composition and a similar powder diffraction pattern. Moreover, during this work, we also solved a hitherto unknown crystal structure, Ce_3_Ni_4_Si_2_ (formerly CeNi_1.3_Si_0.7_). Interestingly, all phases studied in this work are part of some homologue series, one of which was recently discovered in the La–Ni–Si system with the general formula of RE_*n*(*n*+1)+*x*_Ni_*n*(*n*+5)+*y*_Si_(*n*+1)(*n*+2)–*z*_.^14^

Homologue series in solid-state compounds have been widely known and studied, among which Ruddlesden–Popper (RP) phases with general formula A_*n*+1_B_*n*_X_3*n*+1_ are well represented in various systems.^15,16^ The RP phase is built from alternating ABX_3_ slabs with perovskite structure and AX slabs with rock salt structure. Numerous combinations of A, B, and X elements, together with different numbers of *n*, resulted in a large number of compounds in this series.^9^ Replacing the rock salt blocks with different slabs may result in different homologue series such as the Dion–Jacobson phase^17^ and the Aurivillius phase.^18^ Moreover, modifying the inorganic cations with organic cations such as alkyl ammonium gave rise to a new class of compounds known as hybrid organic–inorganic compounds with tunable lattice parameter and dimensionality.^19,20^

In the family of intermetallic compounds, a homologue series with the same formula as the RP phase exists in the RE–Co-Ge system: (RE_*n*+1_Co_*n*_Ge_3*n*+1_).^21^ Despite the similarity in the composition, the building blocks of this series are completely different from those of the RP phase, where the structure is formed from BaNiSn_3_, AuCu_3_, and AlB_2_-type slabs. In the RE–Ni-Si system, the only previously known homologue series was the RE-rich series RE_(*n*+1)(*n*+2)_Ni_*n*(*n*–1)+2_Si_*n*(*n*+1)_, which derived from the Fe_2_P-type structure.^9,22^ The structure is characterized by a small *c*/*a* ratio of the hexagonal cell, where its value decreases with an increasing *n*. The nickel-rich analogue of this series are well-known only among the RE-TM-Pn systems (Pn = P and As).^9^ Different combinations of divalent metals were also known to form quaternary compounds with similar structures, such as Ba_2_Yb_0.88_Mg_11.12_Si_7_, Ba_5_Yb_2.26_Mg_16.73_Si_12_, and Ba_20_Yb_4.70_Mg_61.30_Si_43_.^23^ Such combinations provide the possibility for tuning the DOS, thus providing a pathway to control the electrical transport properties. Moreover, the complex structure of these phases may lead to low thermal conductivity, which is beneficial for thermoelectric applications.

In general, detailed knowledge on the crystal structure and the building block of the homologue series provides important information to design materials with specific properties. Moreover, information about the phase equilibria serves as valuable guidance to synthesize the desired materials. Therefore, in this work, we present a detailed study on the crystal structure, formation, and stability of phases forming homologue series in the Ce–Ni–Si system, as well as their structural relationship.

## Experimental Methods

Samples were synthesized by the fusion of all starting materials via Ti-gettered argon arc-melting on a water-cooled copper hearth. Nickel plates and pieces (99.9 mass%, Alpha Ventron, D), silicon pieces (99.999 mass%, Alpha Ventron, D), and cerium pieces (with freshly cleaned surfaces, 99.8 mass%, Auer Remy, D) were used as starting materials. Samples were melted three times to ensure homogeneity. Heat treatments were performed at various durations and temperatures to achieve equilibrium. For heat treatments at 800 °C, samples were directly vacuum sealed in quartz ampules, while for annealing at higher temperatures (≥900 °C), Al_2_O_3_ crucibles coated with boron nitride were additionally used to avoid reaction with the quartz tube, particularly for samples in the Ce-rich region (>30 at. % Ce). After heat treatments, samples were subsequently quenched in cold water.

All samples, both as cast and annealed, were analyzed by powder X-ray diffraction and electron probe microanalysis (EPMA). X-ray powder diffraction was performed using Ge-monochromated Cu–Kα_1_ radiation in a Guinier–Huber image plate recording system. Phase and structural analyses via Rietveld refinements were performed with the program FullProf.^24^ Precise lattice parameters were calculated employing Ge or Si as an internal standard (*a*_Ge_ = 0.565791 nm; *a*_Si_ = 0.543107 nm).

Single crystals for structural analysis were selected from crushed annealed alloys. Selected specimens were mounted on glass capillaries followed by preliminary inspections on an AXS D8-GADDS to ensure high crystal quality, lattice parameters, and crystal symmetry. Suitable single crystals were then measured at room temperature on a Bruker APEX II diffractometer equipped with a CCD area detector and an Incoatec Microfocus Source IμS (30 W, multilayer mirror, Mo Kα; λ = 0.071069 nm; detector distance of 4 cm; full sphere; 2° < 2θ < 70°). Orientation matrices and unit cell parameters were derived using the Bruker APEX II software suite.^25^ Besides the general treatment of absorption effects using the multiscan technique (SADABS; redundancy of integrated reflections >8), no additional absorption corrections were performed because of the rather symmetric shape and small dimensions of the investigated specimens. The structures were solved by direct methods using program SIR-92^26^ and refined with program SHELXL-97^27^ within the WinGX package.^28^ The crystal structure data were subsequently analyzed with PLATON^29^ for missing symmetry and standardized with program STRUCTURE-TIDY.^30^

Samples for the EPM analyses were first embedded in conductive resin, followed by standard metallographic procedures of grinding and polishing. The quality of polishing and the preliminary investigation of the microstructure were carried out using light optical microscopy. Detailed microstructure and compositions were investigated by EPM analyses on a Zeiss Supra 55 VP instrument at 20 kV equipped with an energy-dispersive X-ray detector.

A Philips CM12 STEM transmission electron microscope (TEM) operated at 120 kV with an EDAX EDX analyzer was employed to obtain information about the crystal symmetry and lattice parameters of selected phases. The samples for the TEM study were prepared in the form of thin lamellae (lateral dimensions about 10 × 7 μm^2^) using a focused ion beam (FIB) technique in a Tescan LYRA 3 XMU FEG/SEM × FIB scanning electron microscope (SEM).

For melting point measurements, alloy specimens were placed in BN-coated Al_2_O_3_ crucibles in Netzsch 404 Pegasus DSC equipment and were run under a stream of 6 N argon with heating rates of 5 K/min. Calibration was made in the temperature range from 300 to 1450 °C against the melting points of pure standard metals supplied by Netzsch to be within ±1 °C.

No uncommon hazards are noted in all experiments described above.

## Results and Discussion

### Formation, Stability, and Phase Relations

**Figure 1:**
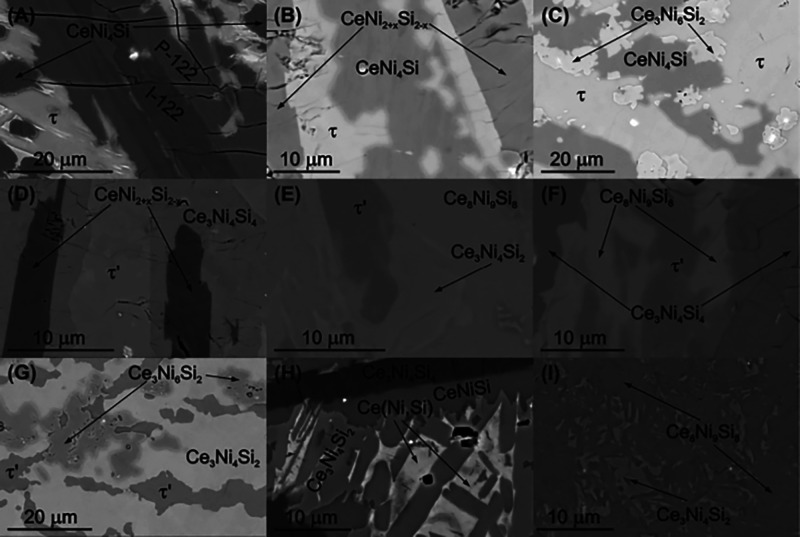
Selected micrograph of as-cast (A, H) and annealed alloys at 800 °C (B–G, I) in the Ce–Ni–Si system.

A new phase Ce(Ni_1–*x*_Si_*x*_)_3_ with composition of Ce∼25Ni∼45Si∼30 (*x* = 0.4, labeled as τ) was first observed in an as-cast sample with nominal composition of Ce20Ni42Si38 during our previous investigation on the BaAl_4_ derivative phases in the ternary and quaternary {La,Ce}-(Ni,Zn)-Si systems.^10^ The amount of this phase in the as-cast state increases as the sample composition shifts toward the Ni side at constant Ce content. Based on the microstructure of an as-cast sample with composition of Ce22.8Ni52.3Si24.9 (Figure 1A), it can be concluded that this new phase forms incongruently. Large primary crystals of CeNi_2+*x*_Si_2*–x*_ (ThCr_2_Si_2_-type: I-122) dominate the sample in the as-cast state, followed by a peritectic crystallization of CeNi_2+*x*_Si_2–*x*_ (CaBe_2_Ge_2_-type: P-122) and the τ phase. The large CeNi_2+*x*_Si_2–*x*_ grains along with the high melting point (*T*_m_ = 1615 °C^31^) posed a problem during equilibration, particularly at 800 °C due to low diffusion kinetics at the mentioned temperature. Synthesis of the τ phase, however, was rather straightforward, yielding a nearly single-phase sample after a week of annealing at 800 °C. Longer annealing time did not improve the sample quality significantly; *i.e*., primary CeNi_2+*x*_Si_2–*x*_ grains are still present even after annealing for 1 month. Annealing at higher temperatures, *e.g.,* 1000 °C, provides better sample quality in shorter time.

According to the 800 °C isothermal section of the Ce–Ni–Si system constructed by Bodak and Gladyshevskii,^3^ the only Ni-rich (≥40 at. % Ni) ternary phase with a Ce concentration close to 25 at. % was Ce_3_Ni_6_Si_2_ (27.3 at. % Ce). The latest revision of the isothermal section by Morozkin et al.^12^ introduced some new phases, including a phase with composition close to our τ phase but with unknown structure. The newly discovered τ phase and its powder diffraction data do not match any Ce–Ni–Si compounds in Pearson’s Crystal Data,^9^ suggesting that it is a novel phase in the system.

Investigation of the homogeneous region of the τ phase revealed an interesting discovery. In the beginning, it looks like this new phase has a relatively constant Si content of ∼32 atom % while the Ce and Ni contents vary from 23.9 to 27.0 at. % Ce and from 45.3 to 42.8 atom % Ni, respectively. A nearly single-phase sample in the Ce-rich side of the homogeneity region of this phase revealed a different X-ray powder diffraction pattern compared to that of the Ce-poor side, suggesting the existence of a different phase (labeled as τ′). A closer inspection on the homogeneity region of this phase indicated that the structural change from τ to τ′ occurs at a Ce concentration of ∼26.0 at. %.

The exact point of transformation, however, cannot be determined reliably as the composition difference between Ce-rich τ and Ce-poor τ′ phase is within the accuracy of the EPM analysis. The composition of the τ′ phase is close to the new unknown phase introduced in Morozkin et al.’s work.^12^ Surprisingly, they did not observe the τ phase during their reinvestigation of the Ce–Ni–Si system.^12^

The Ce-poor composition of the τ phase (23.9 at. % Ce) is in equilibrium with CeNi_2+*x*_Si_2–*x*_ (ThCr_2_Si_2_-type) and CeNi_4_Si (YNi_4_Si-type), while Ce-richer compositions form a three-phase equilibrium with CeNi_4_Si (YNi_4_Si-type) and Ce_3_Ni_6_Si_2_ (own type) (see Figure 1B,C). Figure 1D–G represents the phase fields around τ′. The vertex of the three-phase equilibrium with Ce_3_Ni_4_Si_4_ (U_3_Ni_4_Si_4_-type) and CeNi_2+*x*_Si_2–*x*_ (ThCr_2_Si_2_-type) seems to correspond with the lowest Ce content of this phase (∼26.0 at. % Ce) (Figure 1D). Ce-rich compositions of τ′ form equilibria with Ce_3_Ni_4_Si_4_ (U_3_Ni_4_Si_4_-type), Ce_3_Ni_4_Si_2_ (formerly CeNi_1.3_Si_0.7_, own type), and Ce_8_Ni_9_Si_8_ (unknown).

Single crystals of the τ phase could be obtained easily from any single-phase sample after annealing at 1000 °C for at least 1 week, while single crystals of τ′ could not be obtained with the same method. In fact, various attempts were made to obtain single crystals of τ′ but failed.

Moving toward the Ce-richer region, the phases around 33.3 at. % Ce seem to be consistent with previous reports such as from Bodak and Gladyshevskii^3^ and Morozkin et al.,^12^ with an exception of the existence of a new ternary compound Ce_8_Ni_9_Si_8_ (structure unknown). The composition of Ce_8_Ni_9_Si_8_ is close to that of CeNiSi (LaPtSi-type); however, the XRPD pattern of samples containing this phase could not be indexed with the unit cell of CeNiSi. At 800 °C, this phase possesses a rather small homogeneity region originating from Ni–Si exchange from 34.9 to 36.5 at. % Ni at a constant Ce concentration of ∼32 at. %. The chemical formula corresponds to an ideal composition of Ce32Ni36Si32. Based on the microstructure of as-cast samples in this region, Ce_8_Ni_9_Si_8_ is likely formed via a peritectoid reaction, which usually has a slow reaction rate. This phase is not observed in the as-cast state; instead, CeNiSi (LaPtSi-type) is frequently observed in as-cast samples from this region (Figure 1H, overall composition Ce32.8Ni36.5Si30.8). In this region, Ce_3_Ni_4_Si_4_ dominates the primary crystallization field, followed by the peritectic formation of CeNiSi (LaPtSi-type). Samples containing Ce_8_Ni_9_Si_8_ and Ce_3_Ni_4_Si_2_ are often characterized with fine microstructures after annealing at 800 °C (see Figure 1I). In contrast to Ce_8_Ni_9_Si_8_, Ce_3_Ni_4_Si_2_ is always observed either in the as-cast or in the annealed state. At 800 °C Ce_3_Ni_4_Si_2_ is a point compound with a negligible homogeneity region.

**Figure 2:**
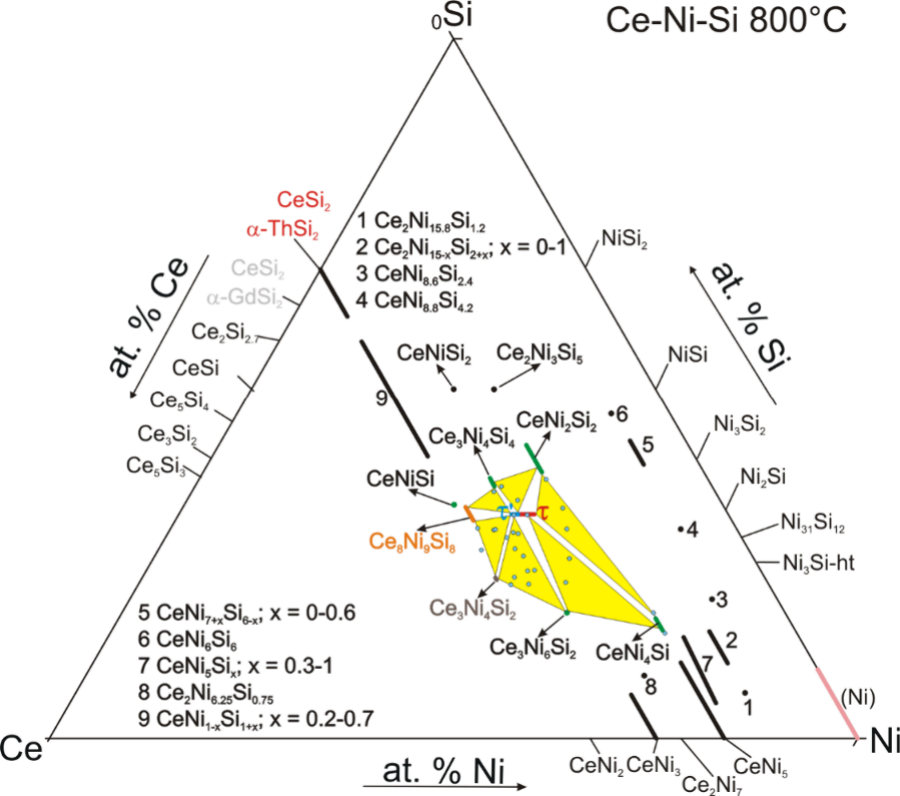
Revised partial isothermal section of Ce–Ni–Si system at 800 °C around phases τ and τ′. Other phases and their homogeneity range in the region of ≤33.3 at. % Ce were taken from Morozkin et al.^12^

Based on the new experimental results, it is necessary to revise the isothermal section of the Ce–Ni–Si system at 800 °C, particularly in the region surrounding τ and τ′ (see Figure 2). With the existence of τ and τ′, several new three-phase regions are introduced. Moreover, in the presence of Ce_8_Ni_9_Si_8_, phase fields related to CeNiSi are now connected to Ce_8_Ni_9_Si_8_. Details of the phase composition from each phase field are listed in Table 1.

**Table 1:** EPMA composition of samples and phases forming three-phase equilibria

sample composition (at. %)				phase composition (at. %)		
Ce	Ni	Si	phases	Ce	Ni	Si
22.4	51.5	26.1	CeNi_4_Si	16.8	67.8	15.4
			Ce_3_Ni_6_Si_2_	26.9	53.8	19.3
			τ	25.3	42.6	32.1
20.8	47.3	31.9	CeNi_2_Si_2_	19.9	39.4	40.7
			CeNi_4_Si	16.9	66.4	16.7
			τ	23.9	43.4	32.7
27.9	43.5	28.6	Ce_3_Ni_4_Si_2_	33.2	44.0	22.8
			Ce_3_Ni_6_Si_2_	27.4	54.6	18.0
			τ′	26.8	41.3	31.9
26.0	38.3	35.7	Ce_3_Ni_4_Si_4_	27.4	36.2	36.4
			CeNi_2_Si_2_	20.1	39.6	40.3
			τ′	26.1	41.5	32.4
30.6	39.3	30.1	τ′	26.9	41.0	32.1
			Ce_8_Ni_9_Si_8_	31.9	36.5	31.6
			Ce_3_Ni_4_Si_2_	33.1	43.9	23.0
27.4	36.9	35.7	τ′	27.0	40.6	32.4
			Ce_3_Ni_4_Si_4_	27.2	36.2	36.6
			Ce_8_Ni_9_Si_8_	32.2	34.9	32.9

### Crystal Structures

#### Crystal Structure of τ-Ce_20+*x*_Ni_36+*y*_Si_30–*z*_—A New Member of the Homologue Series RE_*n*(*n*+1)+*x*_Ni_*n*(*n*+5)+*y*_Si_(*n*+1)(*n*+2)–*z*_ (*n* = 4)

**Figure 3:**
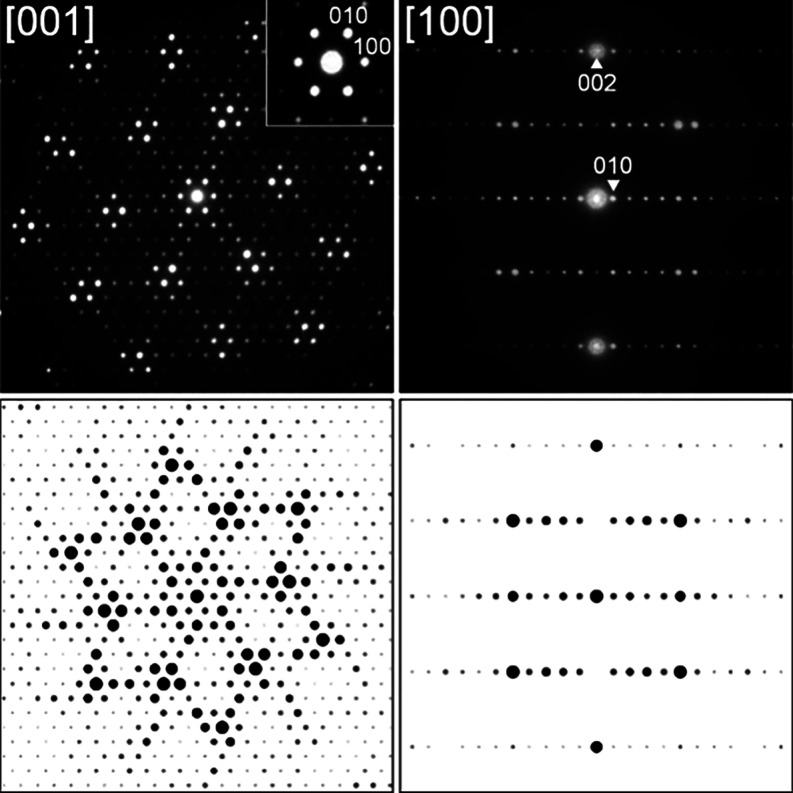
Selected area electron diffraction patterns on the annealed alloy Ce24.6Ni43.5Si31.9 along the [001] and [100] directions (top) and their corresponding simulations (bottom).

A sample with nominal composition of Ce25Ni45Si30 showed nearly single-phase conditions after annealing at 1000 °C for 1 week. The average phase composition obtained from the EPM analysis was Ce24.6Ni43.5Si31.9. A suitable single crystal was then selected from this sample, and a primitive hexagonal unit cell was indexed (*a* = 2.07155(1) nm and *c* = 0.39989(1) nm). Searching for compounds with similar unit cell parameters in Pearson’s Crystal Data^6^ showed that the XRPD pattern of Ce24.6Ni43.5Si31.9 resembles that of the Sm_10_Ni_20.8_P_15_ isotype. Figure 3 shows the two key SAED patterns of alloy Ce24.6Ni43.5Si31.9 from which the single crystal was selected, together with the simulated diffraction patterns calculated using software JEMS.^32,33^ No superstructure reflection was detected, thus confirming the XRSC data.

Final refinement with anisotropic displacement parameters for all atoms except those with small occupancies (less than 0.3) converged to an *R*_F_ value of 0.0482 with the refined composition of Ce24.5Ni43.8Si31.7 (in at. %), finally matching well with the average EPMA composition (for details, see Table 2 and Table S1). The refined formula of the unit cell, Ce_21.8_Ni_39.0_Si_28.2_, corresponds to *n* = 4 in the general structure series RE_*n*(*n*+1)+*x*_Ni_*n*(*n*+5)+*y*_Si_(*n*+1)(*n*+2)–*z*_ with *x* = 1.8, *y* = 3.0, and *z* = 1.8.

**Table 2:** Crystallographic Data for La_20+*x*_Ni_36+*y*_Si_30–*z*_ and Ce_20+*x*_Ni_36+*y*_Si_30–*z*_ SC-1, Ce_20+*x*_Ni_36+*y*_Si_30–z_ SC-2^a^

**compound**	**La**_**20+*x***_**Ni**_**36+*y***_**Si**_30–*z*_	**Ce**_**20+*x***_**Ni**_**36+*y***_**Si**_30–*z*_**SC-1**	**Ce**_**20+*x***_**Ni**_**36+*y***_**Si**_30–*z*_**SC-2**
CCDC	2321442	2321440	2321441
EMPA composition [at. %]	La_25.9_Ni_43.7_Si_30.3_	Ce_24.5_Ni_43.5_Si_31.9_	Ce_24.5_Ni_43.5_Si_31.9_
refinement composition [at.%]	La_25.6_Ni_42.2_Si_32.2_	Ce_24.5_Ni_43.8_Si_31.7_	Ce_24.9_Ni_43.4_Si_31.7_
refinement composition	La_22.4_Ni_36.9_Si_28.2_	Ce_21.8_Ni_39.0_Si_28.2_	Ce_22.2_Ni_38.5_Si_28.5_
formula weight [g/mol]	6069.28	6135.58	6170.71
space group	*P*6_3_/*m*; No. 176	*P*6_3_/*m*; No. 176	*P*6_3_/*m*; No. 176
structure type	Sm_10_Ni_20.8_P_15_ variant	Sm_10_Ni_20.8_P_15_ variant	Sm_10_Ni_20.8_P_15_ variant
2θ range [°]	8.12 < 2θ < 60	8.19 < 2θ < 60	8.21 < 2θ < 60
*a* [nm]	2.09037(2)	2.07156(2)	2.06781(3)
*c* [nm]	0.41231(1)	0.39990(1)	0.39965(1)
*Z*; volume [nm^3^]	1; 1.5602(1)	1; 1.4862(1)	1; 1.4799(1)
ρ [g/cm^3^]	6.46	6.86	6.93
μ [mm^–1^]	26.98	29.36	29.48
*F*(000)	2705	2751	2765
index ranges	–34 ≤ *h* ≤ 34	–34 ≤ *h* ≤ 34	–34 ≤ *h* ≤ 33
	–34 ≤ *k* ≤ 34	–34 ≤ *k* ≤ 34	–34 ≤ *k* ≤ 33
	–6 ≤ *l* ≤ 6	–6 ≤ *l* ≤ 6	–6 ≤ *l* ≤ 6
measured reflections	61,218	54,472	53,744
unique reflections	1636 F_o_ > 4σ(F_o_) of 1707	1598 F_o_ > 4σ(F_o_) of 1641	1578 F_o_ > 4σ(F_o_) of 1626
number of parameters	115	111	111
*R*_int_; *R*_sigma_	0.0459; 0.0131	0.0403; 0.0132	0.0347; 0.0112
*R*_F_; wR2	0.0379; 0.0714	0.0482; 0.0938	0.0495; 0.0961
GOF	1.295	1.318	1.372
extinction (Zachariasen)	0.00052(2)	0.00053(3)	0.00046(3)
residual electron density; max; min in [electrons/nm^3^] × 1000	2.46; −2.72	2.32; −4.85	2.54; −3.04

a Measurements were performed on an APEX II diffractometer at room temperature with Mo K_α_ radiation.

Coordination polyhedra for all atoms excluding those in the vicinity of the screw axis are presented in Figure S3 of the Supporting Information. In general, the cerium atoms are coordinated by nickel and silicon atoms in a bicapped hexagonal prism environment (CN = 14), except for the Ce1 and Ce4 atoms, which possess tetra-capped (CN = 16) and normal hexagonal prism coordination (CN = 12), respectively.

**Figure 4:**
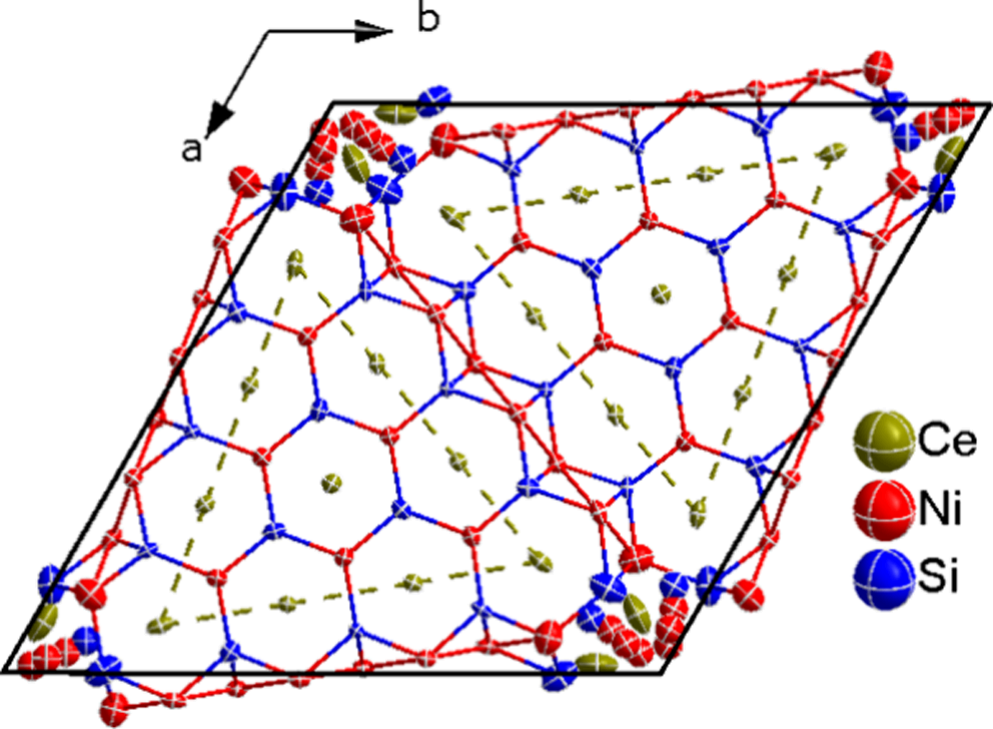
Crystal structure projection of τ-Ce_20+*x*_Ni_36+*y*_Si_30–*z*_ along the *c* axis. Atoms are displayed with their anisotropic thermal displacement ellipsoids (at 90% probability level) as derived from X-ray single-crystal refinement (Table 2).

Figure 4 shows the crystal structure of τ-Ce_20+*x*_Ni_36+*y*_Si_30–*z*_, where the Ce4 atom is surrounded by a triangular arrangement of four capped hexagonal prisms formed by the other cerium atoms (indicated by dashed green lines in Figure 4). The number of hexagonal prisms forming the triangle corresponds to *n* = 4 in the general formula for this compound.

The interatomic distances between Ce and its first coordination sphere, which consists of Ni and Si atoms, range from 0.300 to 0.314 nm: typical values for Ce–Ni and Ce–Si distances. No direct homoatomic contacts between fully filled sites were detected, except for Ni–Ni contacts.

**Figure 5:**
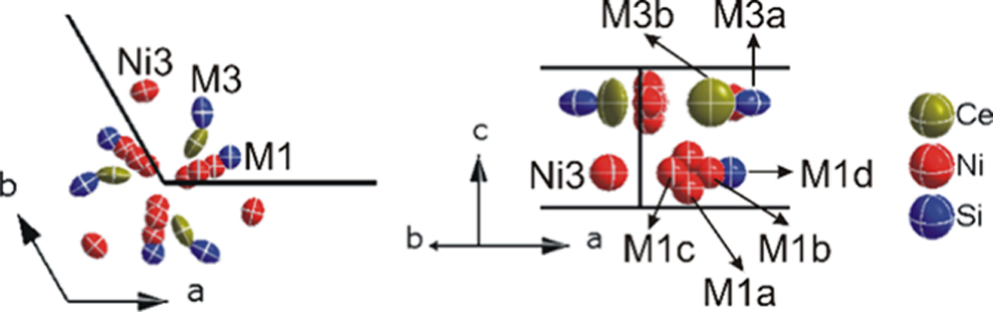
Disorder in τ-Ce_20+*x*_Ni_36+*y*_Si_30–*z*_ near the 6_3_-screw axis along the *c*-axis (left) and a–b axes (right).

The disorder near the 6_3_-screw axis is presented in Figure 5. In general, there are two groups of disordered sites with split positions, denoted as M1 (M1a to M1d) and M3 (M3a to M3d). M1 sites consist of Ni and Si atoms with small occupancies, while the M3 sites are filled with Ce and Si with rather high occupancy, with their sum occ(Ce+Si) in M3 close to unity (for details, see Table S1). Such disorder with split positions is also observed in the La-analogue phases with *n* = 3–6.^14^ The homogeneity range of this phase is related to the site occupancy in the disordered region. Refinement on another single crystal of this compound obtained from the Ce-rich sample with smaller unit cell volume resulted in the same atomic arrangement but with different occupancies. In the Ce-rich crystal, the occupancy of the Ce atom in the M3 site is increased while the Ni occupancy in the M1 site is lowered. The same situation is also observed for the La-analogue phase, where we also obtained a single crystal with a smaller unit cell volume. The refined composition (La_22.4_Ni_36.9_Si_28.2_) is slightly lower in nickel and richer in La compared to the result of Grilli et al.^14^

**Figure 6:**
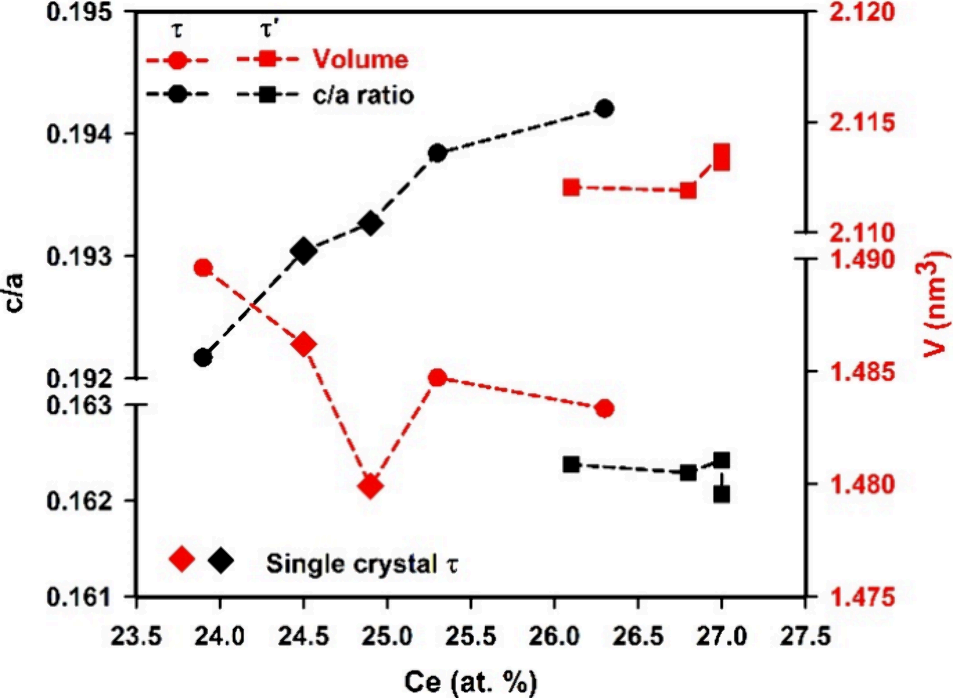
Unit cell volume and *c*/*a* ratio of τ and τ′-phase with different Ce contents.

Further detailed analysis of the lattice parameters is shown in Figure 6. Polycrystalline alloys revealed that instead of the unit cell volume, the *c*/*a* ratio of the τ-phase shows a monotonous increase with increasing Ce content. The unit cell volume shows a similar tendency except for the data of SC-2, which show a significant drop when compared to other samples. The reason behind the drop is still unclear; however, it is important to keep in mind that EPM analysis is not accurate enough to distinguish alloys with close compositions.

It is also important to note that the single crystals were grown at 1000 °C while the polycrystalline alloys were treated at 800 °C. The difference in the temperature could result in a different defect arrangement, which could affect the lattice parameters.

It is worth noting that the atomic arrangement and composition in the disordered area of τ-Ce_20+*x*_Ni_36+*y*_Si_30–*z*_ slightly differ from that of the La analogue. In both cases, all atoms forming the hexagonal prism are free from disorder, except for those forming the RE1-centered hexagonal prism, which is located closest to the origin. In the Ce compound, only one of the Si atoms possesses some disorder (M3 site), while in the La compound, the disorder is present on the Ni and Si atoms. Moreover, in the La compound, the RE atoms in the M3 site exhibit more disorder. The La atom in the M3 site is split into two sites: M3c (6*h* site) and M3d (12*i* site) with occupancies of 0.14(1) and 0.12(1), respectively. Such a situation is not observed in the Ce compound, where the M3-Ce atom only stays at the 6*h* site with an occupancy of ∼0.3. Rietveld refinement on a nearly single-phase sample, however, could not resolve the disorder properly: only sites with sufficiently high electron density, *e.g.,* M3, could be detected and resolved.

#### Crystal Structure of τ′-Ce_30+*x*_Ni_50+*y*_Si_42–*z*_—A New Member of the Homologue Series RE_*n*(*n*+1)+*x*_Ni_*n*(*n*+5)+*y*_Si_(*n*+1)(*n*+2)–*z*_ (*n* = 5)

**Figure 7:**
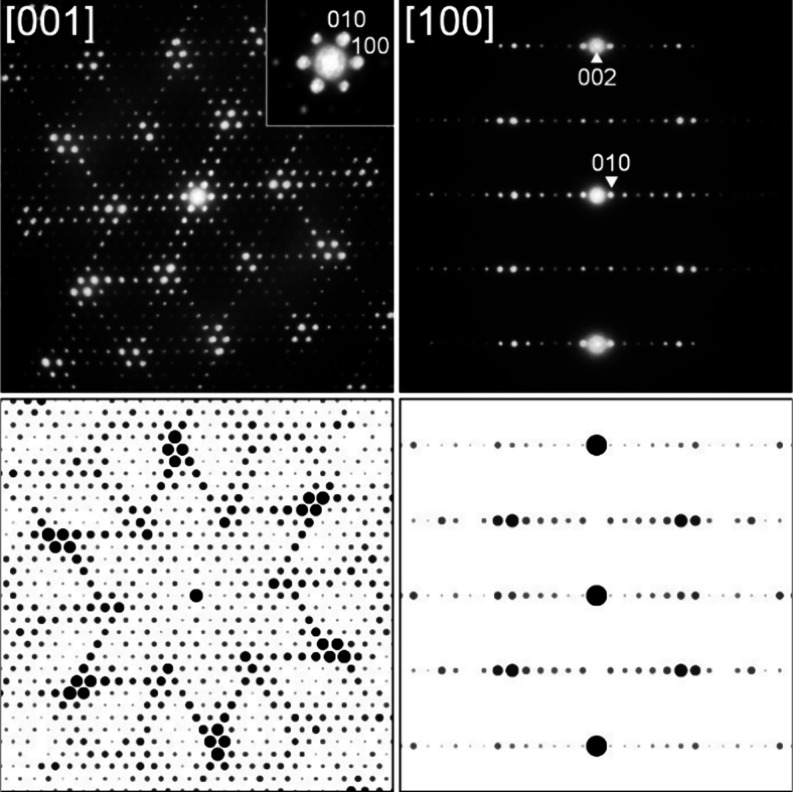
SAED patterns on the annealed alloy Ce26.2Ni42.1Si31.7 along the [001] and [100] directions (top) and their corresponding simulations (bottom).

Attempts to grow a single crystal of this compound were unsuccessful: single crystals selected from crushed alloys with Ce-rich composition Ce26.2Ni42.1Si31.7 always resulted in τ-phase crystals. Interestingly, the bulk XRPD pattern of the sample shows a nearly single-phase τ*′*-phase. Selected area electron diffraction (SAED) on a lamella produced by FIB from an annealed alloy Ce26.2Ni42.1Si31.7 revealed sixfold symmetry along the [001] direction, indicating a hexagonal unit cell (see Figure 7) with *a* = 2.442 nm and *c* = 0.4084 nm.

**Figure 8:**
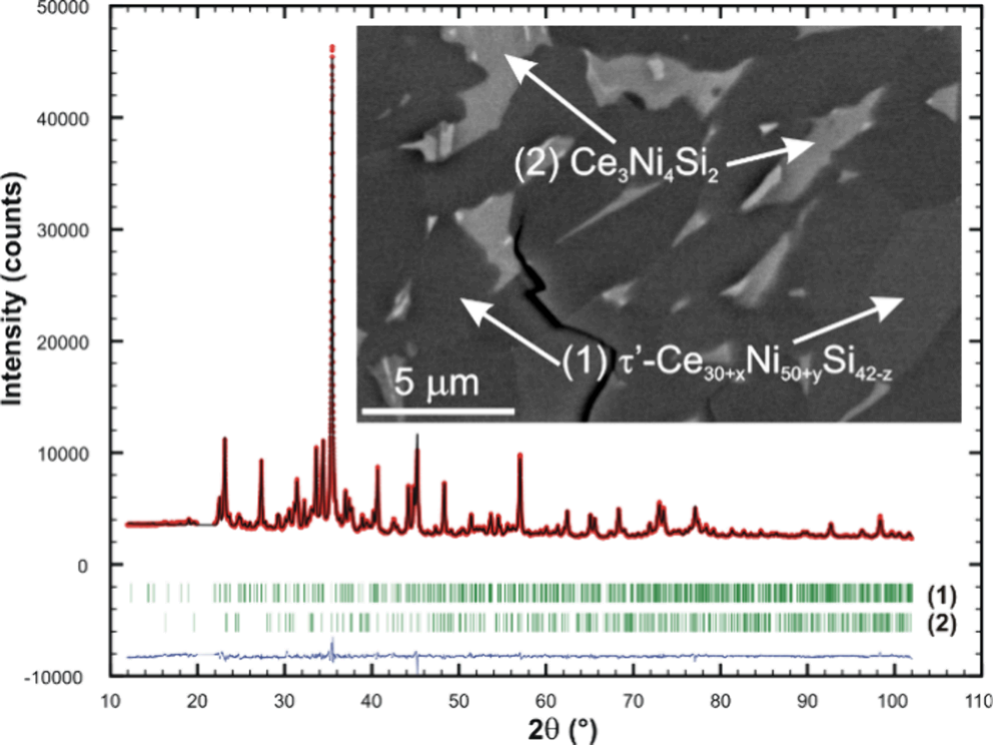
Rietveld refinement of an alloy with composition Ce28.0Ni41.6Si31.4 annealed at 900 °C. Inset shows the micrograph of the sample.

Analyzing another sample with composition Ce28.0Ni41.6Si31.4 annealed at 900 °C (see Figure 8) yielded precise lattice parameters from the X-ray powder diffraction pattern with Si as internal standard for a hexagonal unit cell with *a* = 2.46926(13) nm and *c* = 0.40019(3) nm. Search for compounds with similar lattice constants in Pearson’s Crystal Data^6^ indicated that Tb_15_Ni_28_P_21_ possesses a diffraction pattern corresponding to τ′.^34^

Rietveld refinement using the data set of Tb_15_Ni_28_P_21_ yielded a final *R*_I_ value of 4.1%. It should be noted that a Rietveld refinement using the data set from Grilli et al.^14^ resulted in a practically similar *R*_I_ value; however, some atomic sites in the disordered area could not be refined reliably. In any case, the details of the atomic distribution around the 6_3_-screw axis could not be well resolved from the XRPD data. Details of the Rietveld refinement results are presented in Table 3 and Table S2. The refined unit cell formula Ce_31.5_Ni_48.4_Si_40.3_ (Ce_26.2_Ni_40.3_Si_33.5_ in at. %) agrees reasonably well with the EPMA-derived composition Ce_27.0_Ni_41.3_Si_31.7_, despite the lack of detailed atomic distribution in the disordered area.

**Table 3:** Rietveld Refinement Data of Ce_30+*x*_Ni_50+*y*_Si_42–*z*_^a^

**compound**	**Ce**_**30+*x***_**Ni**_**50+*y***_**Si**_42–*z*_
EMPA composition [at. %]	Ce_27.0_Ni_41.3_Si_31.7_
refinement composition [at. %]	Ce_26.4_Ni_40.5_Si_33.1_
refinement composition	Ce_32.0_Ni_49.1_Si_40.1_
formula weight [g/mol]	8491.79
space group	*P*6_3_/*m*; No. 176
structure type	Tb_15_Ni_28_P_21_ variant
2θ range [°]	10 < 2θ < 100
*a* [nm] (Si standard)	2.46926(13)
*c* [nm] (Si standard)	0.40019(3)
reflections in refinement	974
*Z*; volume [nm^3^]	1; 2.1131(1)
ρ [g/cm^3^]	6.68
number of parameters	66
*R*_I_; *R*_p_; *R*_wp_; *R*_exp_; χ^2^	0.041; 0.0820; 0.0841; 0.00549; 2.23

a Measurements were performed using a Guinier–Huber image plate at room temperature with Cu K_α1_ radiation.

**Figure 9:**
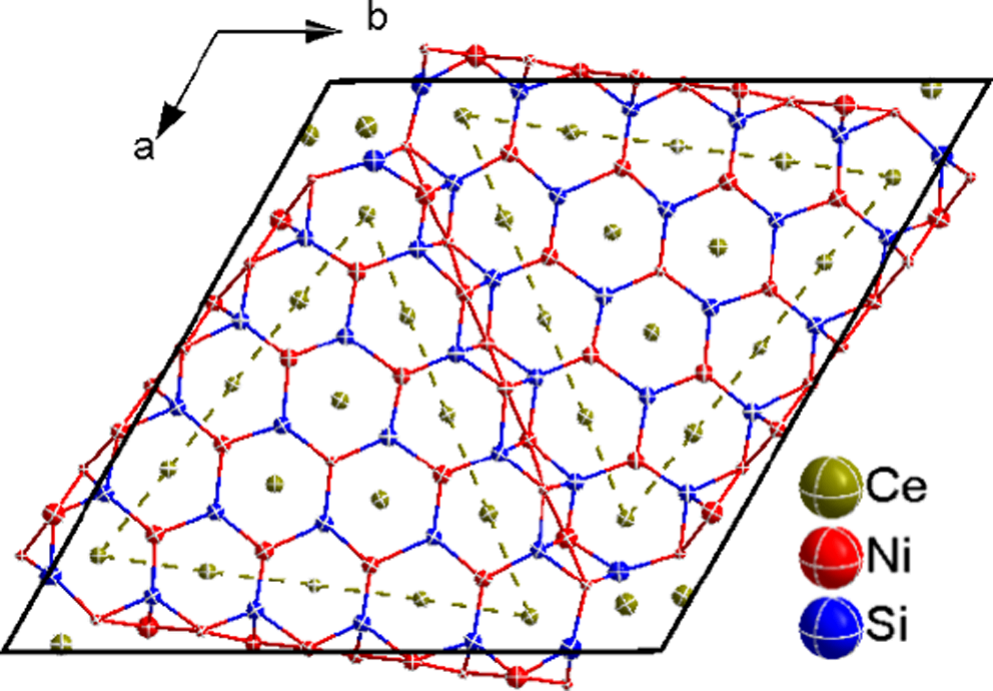
Crystal structure projection of τ′-Ce_30+*x*_Ni_50+*y*_Si_42–*z*_ in the [001] direction. Atoms are displayed with their isotropic thermal displacement ellipsoids (at the 90% probability level) as derived from Rietveld refinement (see Table S2).

The crystal structure of the τ′ phase is shown in Figure 9. This compound can be considered as an enlarged version of the τ phase, where the number of Ce-centered hexagonal prisms forming the triangle is larger (*n* = 5). The enlargement is also associated with an exchange of Ce and Ni, whereas the τ′ phase is slightly richer in Ce as compared to the τ-phase. Despite our inability to elucidate the disorder in detail, the presence of disorder near the origin is also reflected in the shape of the Ce-centered hexagonal prism. The prisms located near the origin possess a more distorted hexagonal shape (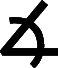 Ni4–Si7–Ni3 = 131° and 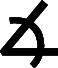 Si1–Ni3–Si7 = 113°) compared with those located far away from the origin, i.e., in the center of the unit cell. Such a situation is not observed in the case of the single-crystal-derived structure of the La-analogue phase and τ phase (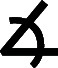 Ni–Si–Ni and 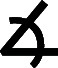
Si–Ni–Si = 120 ± 3°). While EPM analyses gave a hint to a small homogeneity region from ∼26.0 to 27.0 at. % Ce at a constant Si concentration of 32.0 atom %, the unit cell volume as well as *c*/*a* ratio do not vary significantly with different Ce contents (see Figure 6).

In contrast to the La–Ni–Si system for which four members of this series were observed,^14^ only two members were discovered in the Ce–Ni–Si system, with *n* = 4 and 5. Members with *n* = 3 would have a composition close to Ce21.2Ni47.7Si31.1 (in atom %); however, this composition was neither observed in the 800 °C or in the 1000 °C isothermal section of the Ce–Ni–Si system. Instead, we observed a three-phase region containing the Ce-poor τ-phase, CeNi_2+*x*_Si*_x_*, and CeNi_4_Si. The member with *n* = 6 would be located at less than 1 at. % from the composition of τ′, where we also did not observe any indication of its formation, neither from XRPD nor EPMA. Perhaps one would need to look at lower temperature to observe the formation of such members. In this condition, sometimes suitable single crystals are difficult to obtain, and thus combinations of more sophisticated structure elucidation techniques such as HR-TEM, electron diffraction tomography, and synchrotron diffraction should be employed to obtain the detailed crystal structure.

#### Crystal Structure of Monoclinic Ce_3_Ni_4_Si_2_ (Formerly CeNi_1.3_Si_0.7_)—A New Homologue Series

An alloy with the composition of Ce32.6Ni45.0Si22.4 annealed at 800 °C for 14 days showed a two-phase mixture of Ce_3_Ni_4_Si_2_ (major) and Ce_3_Ni_6_Si_2_ (minor). A single crystal selected from this alloy was successfully indexed on a *C*-centered monoclinic unit cell with *a* = 1.54708(3) nm; *b* = 0.58677(1) nm; *c* = 0.74331(1) nm; and β = 102.985(1)°. Refinement in the space group *C*2/*c* revealed a fully ordered structure comprising five atomic positions, four of which in the general positions of 8*f*, assigned as Ce1, Ni1, Ni2, and Si1. An additional Ce atom (Ce2) is in the 4*e* position, resulting in a Wyckoff sequence of f^4^e. Final refinement employing anisotropic atomic displacement parameters for all atoms resulted in a satisfactory *R*_F_ value of 0.0173 with a residual electron density less than ±2000 e^–^/nm^3^. Details of the refinements and atomic positions are provided in Table 4 and Table S3, respectively. The refined composition then corresponds to an empirical formula of Ce_3_Ni_4_Si_2_, which is close to the formula of CeNi_1.3_Si_0.7_, previously reported in the literature,^3,12^ as well with the EPMA-derived composition of Ce33.2Ni43.9Si22.8 (in at. %). Figure S6 shows the Rietveld refinement of a powdered sample from which the single crystal was selected, thus confirming the structure solution from the single crystal. Formation of isotypic La_3_Ni_4_Si_2_ was confirmed via Rietveld refinement (presented in Table 4 and Figure S7) of a powder sample with a nominal composition of La_3_Ni_4_Si_2_ annealed at 650 °C for 2 weeks. The analyzed sample contains La_3_Ni_4_Si_2_ as a major phase together with LaNi_2_Si, La_2_Ni_3_Si_2_, and several small unindexed peaks.

**Table 4:** X-ray Single-Crystal Data for Ce_3_Ni_4_Si_2_ and the Rietveld Refinement Result for La_3_Ni_4_Si_2_^a^

**compound**	**Ce**_**3**_**Ni**_**4**_**Si**_**2**_	**La**_**3**_**Ni**_**4**_**Si**_**2**_
CCDC	2321438	
EPMA composition [at. %]	Ce_33.2_Ni_43.9_Si_22.8_	La_33.2_Ni_43.9_Si_22.8_
refinement composition [at. %]	Ce_33.3_Ni_44.4_Si_22.2_	La_33.5_Ni_44.2_Si_22.3_
formula weight [g/mol]	711.29	707.66
space group	*C*2/*c*; No. 15	*C*2/*c*; No. 15
structure type	own	Ce_3_Ni_4_Si_2_
data collection	APEX II	Guinier–Huber
radiation	MoK_α_	CuK_α1_
2θ range [°]	8.11 < 2θ < 72.7	15 < 2θ < 100
*a* [nm]	1.54708(3)	1.58245(5)
*b* [nm]	0.58677(1)	0.60029(2)
*c* [nm]	0.74331(1)	0.75006(3)
β	102.985(1)°	103.096(1)°
*Z*; volume [nm^3^]	4; 0.6575(1)	4; 0.6940(1)
ρ [g/cm^3^]	7.19	6.78
μ [mm^–1^]	31.76	
*F*(000)	1256	1244
index ranges	–25 ≤ *h* ≤ 25	
	–9 ≤ *k* ≤ 9	
	–12 ≤ *l* ≤ 12	
measured reflections	11,512	
unique reflections	1335 F_o_ > 4σ(F_o_) of 1478	1093
number of parameters	43	54
*R*_int_; *R*_sigma_	0.0442; 0.0251	
*R*_F_; wR2	0.0173; 0.0328	*R*_I_ = 0.0395; *R*_p_ = 0.182; *R*_wp_ = 0.158
GOF	1.064	*R*_exp_ = 0.1147; χ^2^ = 1.89
extinction (Zachariasen)	0.00076(4)	
residual electron density; max; min in [electrons/nm^3^] × 1000	1.10; −1.85	

a Measurements were performed at room temperature.

Coordination polyhedra for all atoms in Ce_3_Ni_4_Si_2_ are presented in Figure S8. Among all atoms, Ce1 and Ce2 possess the highest coordination numbers of 13 and 12, respectively. The coordination polyhedron of Ce1 is rather irregular, whereas that of Ce2 can be described as a bicapped pentagonal prism. It is interesting to note that within the coordination polyhedron of Ce1, there is a Ce1–Ce1 contact with a rather short distance of 0.32893 nm. This distance is interestingly shorter than Ce1–Ni2 distances of 0.33098 and 0.34617 nm. Despite being located rather far away compared to the other Ni2 atoms, those Ni2 atoms are included in the coordination polyhedron since their contributions to the Dirichlet areas are still significant. Further analysis of interatomic distances reveals short Ce1–Ni distances of less than 0.28 nm. In contrast to Ce1, there is no direct Ce–Ce contact involving Ce2. Moreover, no Ce2-X contacts (X = Ni, Si) with distances less than 0.295 nm are detected within the first coordination sphere. Ni1 possesses coordination number 9 in the form of a tricapped trigonal prism in which the trigonal prism is composed of Ce atoms, capped by Ni and Si atoms. The atomic environment surrounding Ni2 and Si atoms takes the shape of distorted bicapped square antiprisms (CN = 10).

**Figure 10:**
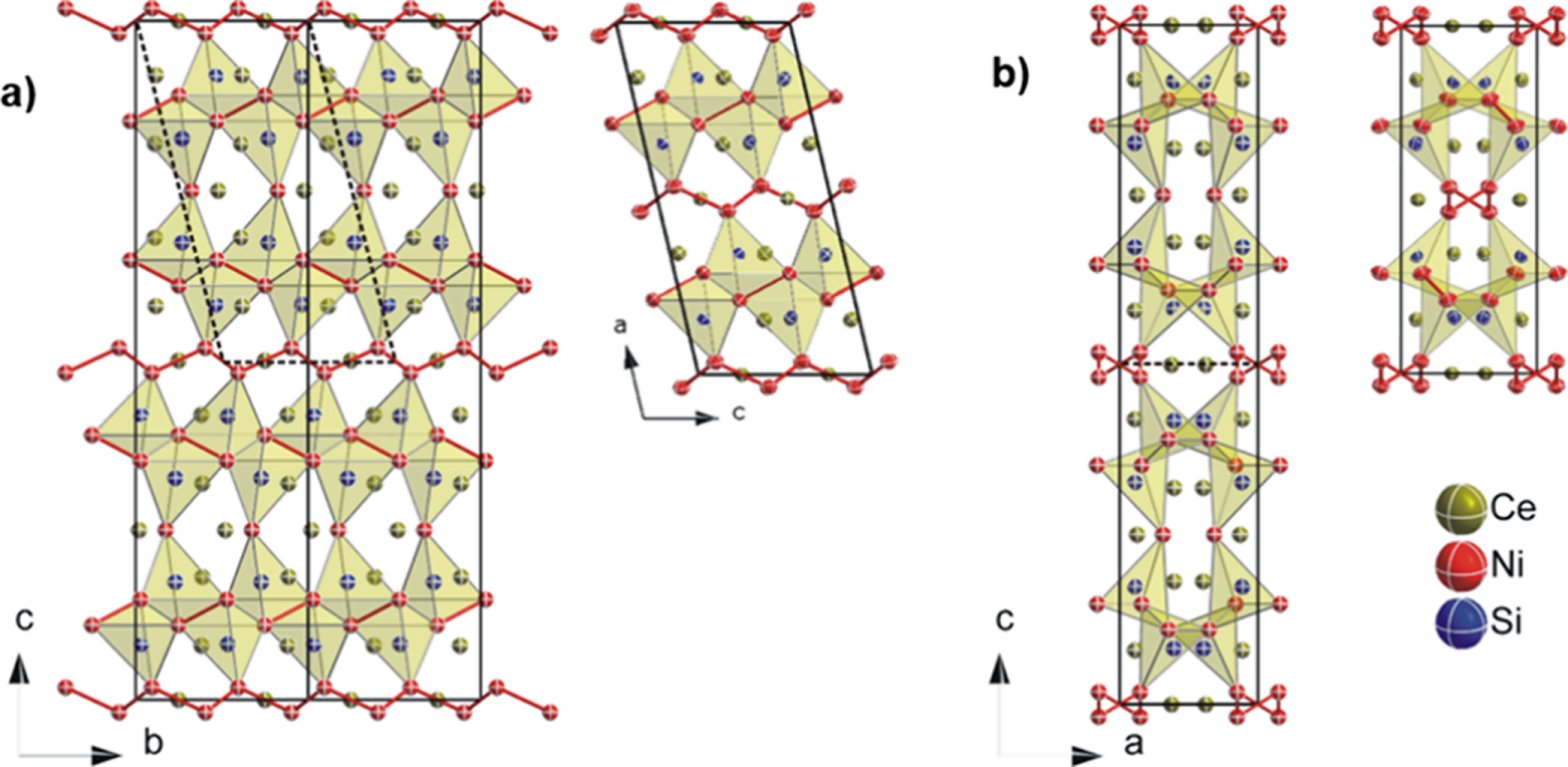
Projection of the crystal structure of (a) Ce_6_Ni_7_Si_4_ along [100] (left) and of Ce_3_Ni_4_Si_2_ along [010] (right); (b) Ce_6_Ni_7_Si_4_ along [010] (left) and of Ce_3_Ni_4_Si_2_ along [001] (right).

Search for binary and ternary Ce–Ni-containing compounds with such interatomic distances in Pearson’s Crystal Data^6^ resulted in 33 entries, one of which, Ce_6_Ni_7_Si_4_ is a neighboring phase in the Ce–Ni–Si system. Ce_6_Ni_7_Si_4_ crystallizes in a primitive orthorhombic unit cell, isotypic with Pr_6_Ni_7_Si_4_ with a unit cell volume of 1.29231 nm^3, 11^, which is almost twice as big than that of Ce_3_Ni_4_Si_2_. The crystal structure of Ce_6_Ni_7_Si_4_ is described as the intergrowth of ThSi_2_- and Y_3_Rh_2_Si_2_-type slabs. Closer inspection and comparison of the crystal structures of Ce_6_Ni_7_Si_4_ and Ce_3_Ni_4_Si_2_ revealed a striking resemblance. The orthorhombic unit cell of Ce_6_Ni_7_Si_4_ is closely related to that of monoclinic Ce_3_Ni_4_Si_2_, with ; *b*_m_ = *a*_o_; *c*_m_ = *b*_o_;  (see Figure 10).

The crystal structures of both compounds consist of two different Ni fragments, namely, a Ni–Ni dumbbell and an infinite zigzag Ni chain along the [010] direction in the orthorhombic setting. The zigzag nickel chains separate the Si-polyhedral block, whereas the Ni–Ni dumbbell is part of the Si polyhedron. For clarity, only atoms within a radius of less than 0.3 nm are drawn for the Si polyhedron. In this case, Si atoms are located at the base of a triangular pyramid formed by 4 Ni atoms.

Ternary compounds containing RE and TMs, in many cases, exhibit a significant homogeneity range. The crystal structures of such compounds are usually characterized by mixed occupancies as well as site vacancies. The compound Ce_6_Ni_7_Si_4_ can be considered as a Ni-deficient variant of Ce_3_Ni_4_Si_2_. In contrast to the usual vacancy/mixed occupancy scheme, removal of some Ni atoms in Ce_3_Ni_4_Si_2_ led to the symmetry change from monoclinic to orthorhombic with doubling of the unit cell volume. Additionally, nickel removal is associated with the removal of a zigzag chain from the structure and formation of corner-sharing Si-polyhedral blocks while the rest of the building blocks stay the same.

The removal of the nickel chain, which corresponds to half a Ni atom per formula unit of Ce_3_Ni_4_Si_2_ (5.56 at. %), results in a 1.73% reduction of the unit cell volume. Such an amount of volume reduction is typical for vacancy formation. For comparison, the change in unit cell volume due to the formation of a nickel vacancy in PrNi_1–*x*_Sb_2_ (HfCuSi_2_-type) is 2.38% from *x* = 0.02 to *x* = 0.38 (9.5 at. %).^35^ The fact that those compounds do not possess a significant homogeneity range is consistent with the symmetry change due to the addition/removal of Ni atoms.

**Figure 11:**
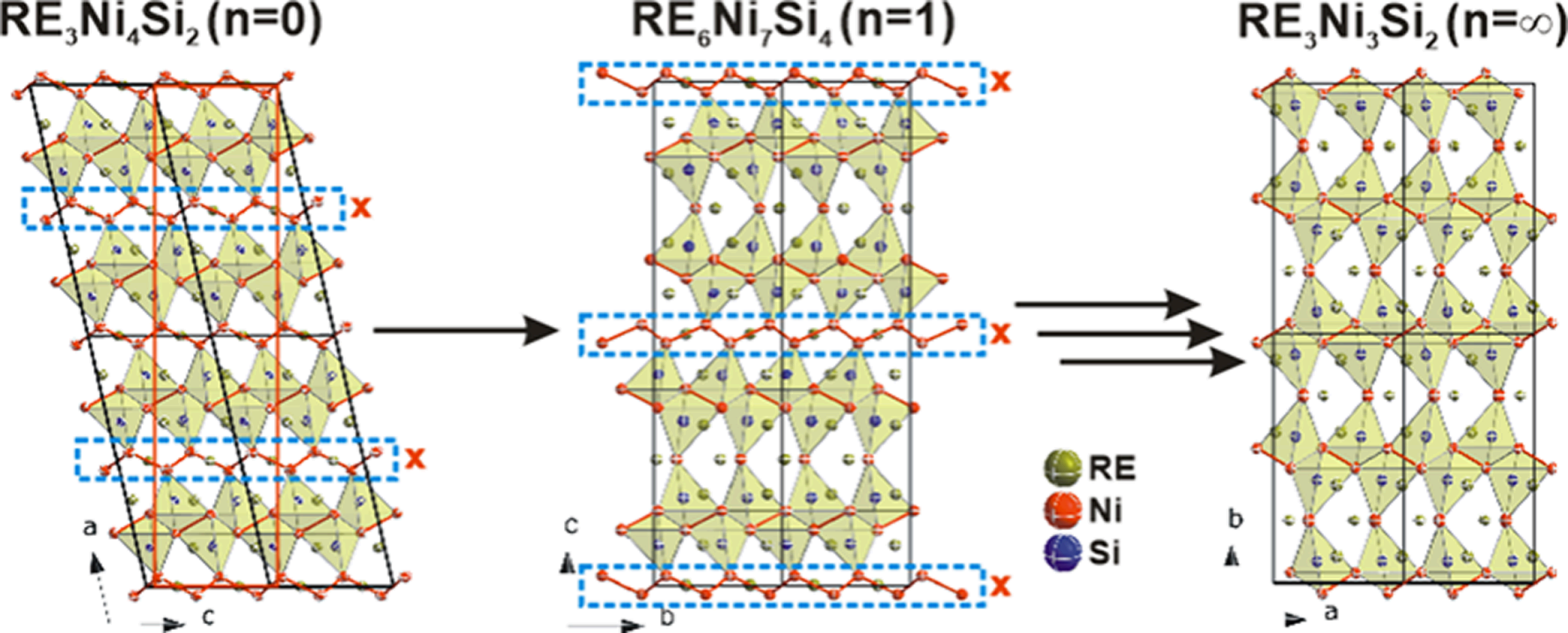
Schematic of homologue series formation by removal of Ni chains. The orthorhombic unit cell of RE_3_Ni_4_Si_2_ is indicated by a red rectangle.

Following the pattern of Ni removal from Ce_3_Ni_4_Si_2_ (see Figure 11), one could hypothesize the formation of a homologue series with formula of Ce_(3×2^*n*^)_Ni_(3×2^*n*^ + 1)_Si_(2^*n*+1^)_; *n* = 0, 1, 2, etc. Compounds in this series would possess a unit cell dimension in orthorhombic setting as follows: *a* ≈ 0.60 nm, *b* ≈ 0.75 nm, and *c* axis length close to double of the previous member. Due to the doubling of the *c* axis, the angle β in the monoclinic setting would get closer to 90° with increasing *n*, thus becoming closer to orthorhombic symmetry. Ce_3_Ni_4_Si_2_ is the first member with *n* = 0, followed by Ce_6_Ni_7_Si_4_ with *n* = 1. Further removal of nickel chains would result in the formation of Ce_12_Ni_13_Si_8_ (*n* = 2) and Ce_24_Ni_25_Si_16_ (*n* = 3) with double and quadruple unit cell volumes compared to Ce_6_Ni_7_Si_4_, respectively. Other members of the series are shown in Table 5. The series would finally converge to the composition Ce_3_Ni_3_Si_2_ (*n* = ∞).

**Table 5:** Members of Homologue Series RE_(3×2^*n*^)_Ni_(3×2^*n*^ + 1)_Si_(2^*n*+1^)_

	**composition**							
	**empirical formula**			**atomic percent**				
***n***	**RE**	**Ni**	**Si**	**RE**	**Ni**	**Si**	*c* **(nm)**^a^	**status**
0	3	4	2	33.3	44.4	22.2	1.612	RE = La,Ce [this work-own type]
1	6	7	4	35.3	41.2	23.5	2.924	RE = La – Pr^11,36^ (Pr_6_Ni_7_Si_4_-type)
2	12	13	8	36.4	39.4	24.2	∼5.5	not discovered
3	24	25	16	36.9	38.5	24.6	∼10.8	not discovered
4	48	49	32	37.2	38.0	24.8	∼21.3	not discovered
								
∞	3	3	2	37.5	37.5	25.0	1.4316	RE = La^36^ (Ce_3_Rh_3_Si_2_-type)

a in orthorhombic setting of RE_6_Ni_7_Si_4_.

While Ce_3_Ni_3_Si_2_ has not been detected in the Ce–Ni–Si system, such a compound was detected recently in the La–Ni–Si system.^36^ Indeed, La_3_Ni_3_Si_2_ (Ce_3_Rh_3_Si_2_-type) shows a close resemblance with Ce_3_Ni_4_Si_2_ and Ce_6_Ni_7_Si_4_. Due to infinite removal of the Ni chains, the only nickel fragment left in La_3_Ni_3_Si_2_ is the Ni–Ni dumbbell. The absence of the zigzag nickel chains and the existence of an infinite corner-sharing Si-polyhedral block indicate that La_3_Ni_3_Si_2_ is the end member of this homologue series. Such situation is akin to the RP phase^15,16^ A_*n*+1_B_*n*_X_3*n*+1_, where a number (*n*) of corner-sharing octahedral BX_6_ layers are separated by AX rock salt layers. The cubic perovskite ABX_3_ is considered as the end member with *n* = ∞, with an infinite number of corner-sharing BX_6_ octahedra. In this case, the AX rocksalt and the BX_6_ octahedron layers are replaced by the zigzag Ni chain and the Si-polyhedral block, respectively. The number of Si-polyhedral blocks sandwiched between two nickel chains is equal to 2^*n*^.

**Figure 12:**
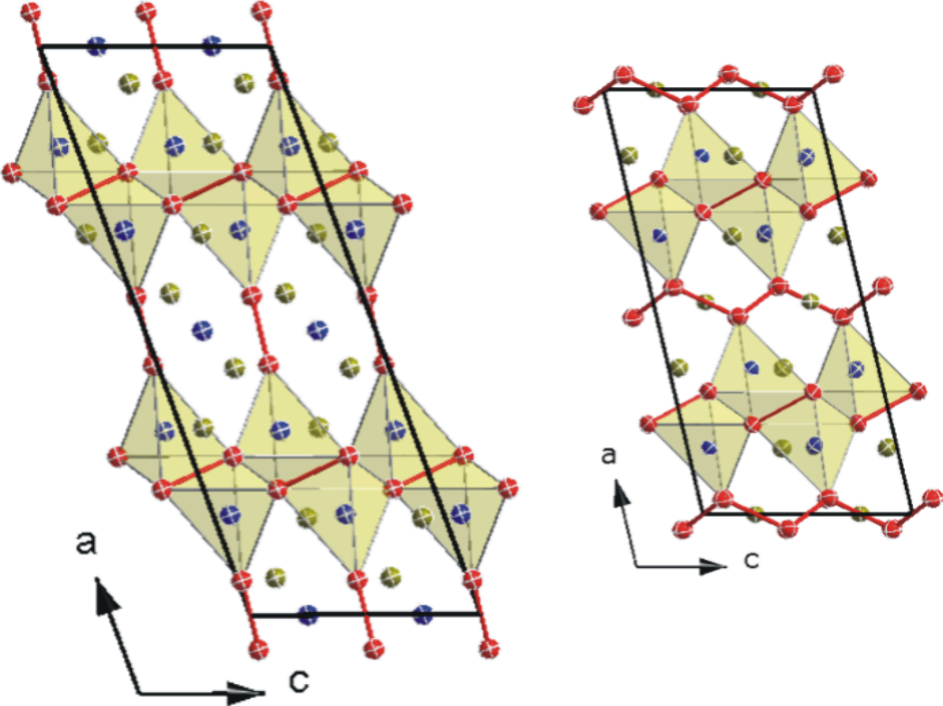
Projection of crystal structures of Ce_4_Rh_4_Si_3_ (left) and Ce_3_Ni_4_Si_2_ (right) along [010].

The crystal structure of Ce_3_Ni_4_Si_2_ is also related to that of Ce_4_Rh_4_Si_3_ (Nd_4_Rh_4_Ge_3_-type).^37^Figure 12 depicts similar block arrangements in Ce_3_Ni_4_Si_2_ and Ce_4_Rh_4_Si_3_. Both structures show the same arrangement of Si-polyhedral blocks but separated by different metal fragments. No infinite metal chains are observed in Ce_4_Rh_4_Si_3_; instead, the Si-polyhedral blocks are separated by Rh–Rh dumbbells. Since neither Ce_3_Rh_4_Si_2_ nor Ce_6_Rh_7_Si_4_ were reported in the Ce–Rh–Si system,^37^ while Ce_3_Rh_3_Si_2_ is present, a different series could exist in the Ce–Rh–Si with Ce_4_Rh_4_Si_3_ and Ce_3_Rh_3_Si_2_ as the first and end members, respectively.

The fact that the end member is not yet observed in the Ce–Ni–Si system could be related to different thermodynamic stabilities or formation preferences with specific RE elements. Members with higher *n* possess higher RE content, and usually the RE-rich region of the phase diagram is dominated by a liquid phase at 800 °C.^38^ Thus, one must explore the lower-temperature region to inspect the formation of those phases. For example, La_3_Ni_4_Si_2_ was reported to be stable below 750 °C^36^ while single crystals of Ce_3_Ni_4_Si_2_ and RE_6_Ni_7_Si_4_ were obtained at 800 °C or higher.^11^ Preference of formation with specific RE atoms can be seen from the fact that there are no reported isotypic RE_3_Ni_4_Si_2_ compounds with RE atoms other than La and Ce. On the other hand, RE_6_Ni_7_Si_4_ is formed with RE = La to Nd. Difficulty in detecting those compounds also arises with *n* > 2, since the compositions would be rather close to each other (within ∼1 at. %). Those members are furthermore expected to exhibit extremely large unit cells (larger than 6 nm).

## Conclusions

Several new phases in the nickel-rich part of the Ce–Ni–Si system have been detected at 800 and 1000 °C: τ-Ce_20+*x*_Ni_36+*y*_Si_30–*z*_ and τ′-Ce_30+x_Ni_50+*y*_Si_42–*z*_ are members of the homologue series RE_*n*(*n*+1)+*x*_Ni_*n*(*n*+5)+*y*_Si_(*n*+1)(*n*+2)–z_, with *n* = 4 and 5. Both compounds form due to Ce–Ni exchange at a Si concentration of ∼32 atom %; τ from 23.9 to ∼26.0 at. % Ce with structural transformation to τ′ at ∼26 atom % Ce. The phases derive from AlB_2_-type forming triangular arrangements of Ce-centered hexagonal prisms with the size of *n*. Both compounds (S.G. *P*6_3_/*m*) are characterized by a large degree of disorder near the 6_3_-screw axis.

Ce_3_Ni_4_Si_2_ (formerly CeNi_1.3_Si_0.7_ with unknown structure) adopts a unique monoclinic structure (*C*2/*c*, *a* = 1.54708(3), *b* = 0.58677(1), *c* = 0.74331(1) nm, β = 102.985(1)°) and forms a new homologue series Ce_(3×2^*n*^)_Ni_(3×2^*n*^ + 1)_Si_(2^*n*+1^)_; *n* = 0,1, ..., ∞ characterized by alternating layers of silicon polyhedral blocks and zigzag nickel chains: Ce_3_Ni_4_Si_2_ (*n* = 0) and Ce_6_Ni_7_Si_4_ (*n* = 1). The La analog phase to Ce_3_Ni_4_Si_2_ was confirmed by Rietveld refinement. Thus, three homologue series exist in the {La,Ce}–Ni-Si systems, and possibly in other RE containing systems. Phase equilibria at 800 °C were revised for the region between 25 and 33 at. % Ce and 20–40 at. % Si.

## Supporting Information Available

Thermal analyses, crystal structure refinements, and table of atomic coordinates, coordination polyhedra, and Rietveld refinements of powder samples (PDF)
