# Supplementary material for: The Ce–Ni–Si System Revisited: More Homologue Compounds?
Source: Inorg Chem. 2024 May 2;63(19):8604–14. doi: 10.1021/acs.inorgchem.3c04594 (PMC11094789; doi:10.1021/acs.inorgchem.3c04594)
Supplement: Supplementary file 1 — ic3c04594_si_001.pdf [file ic3c04594_si_001.pdf]

Supporting Information

The Ce-Ni-Si System Revisited: More Homologue Compounds?

*Fainan Failamani[a,b], Andriy Grytsiv[a], Jiri Bursik[c], Gerald Giester[d], Peter Rogl[a]**

[a]Institute of Materials Chemistry, University of Vienna, Währingerstraße 42, A-1090 Vienna, Austria. *Email: peter.franz.rogl@univie.ac.at

[b]Division of Inorganic and Physical Chemistry, Faculty of Mathematics and Natural Sciences, Institut Teknologi Bandung, Jalan Ganesha 10, 40132 Bandung, Indonesia.

[c]Institute of Physics of Materials, Czech Academy of Sciences, Žižkova 22, 61662 Brno, Czech Republic.

[d]Institute of Mineralogy and Crystallography, University of Vienna, Althanstraße 14, A-1090 Vienna, Austria.

Thermal Analyses

DTA measurements on both phases, τ and τ', in **Figure S1** show an endothermic, broad peak with onset at 1080±1 °C. The broad peak indicates that there are several phase transformations close to

each other around the decomposition of both τ and τ'. Samples annealed at 1100 °C became multiphase; therefore, it is reasonable to assume that the observed thermal effect corresponds to the decomposition temperatures of τ and τ'.

Figure S2 shows the result of a DTA measurement on a nearly single-phase annealed alloy with a composition of Ce32.6Ni45.0Si22.4 revealing two endothermic peaks at 816 °C and 851 °C. A closer inspection on the latter (see the inset in **Figure S2**) shows an additional shoulder with onset at 863 °C. The signal at 816 °C is stronger than the one at 851 °C, indicating that 816 °C is the decomposition temperature of $Ce_3Ni_4Si_2$.

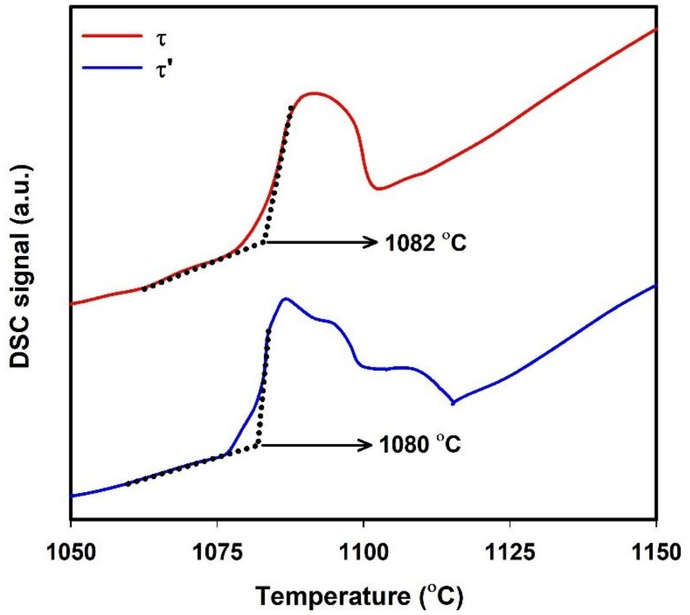

Figure S1. DTA measurements on τ and τ', both annealed at 800 °C.

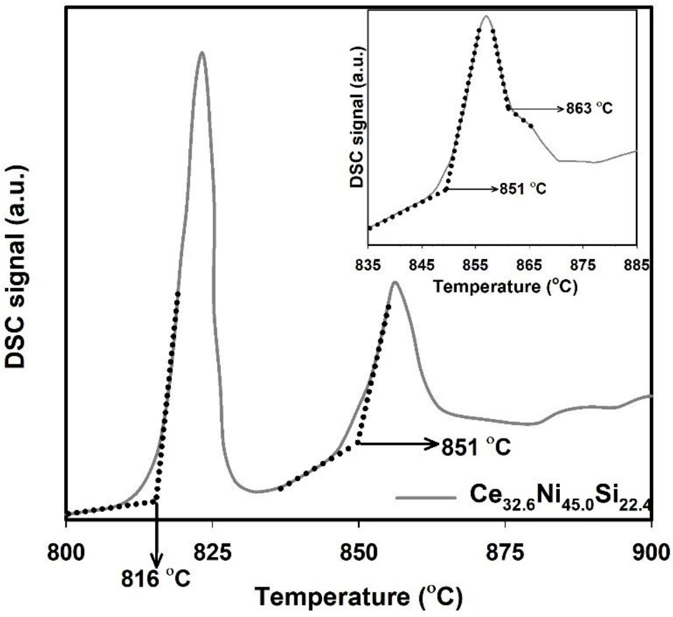

Figure S2. DTA measurements on alloy with composition of Ce32.6Ni45.0Si22.4, annealed at 800 °C.

Crystal Structure

Crystal structure of τ-$Ce_{20+x}Ni_{36+y}Si_{30-z}$ – a new member of the homologue series $RE_{n(n+1)+x}Ni_{n(n+5)+y}Si_{(n+1)(n+2)-z}$ (n = 4)

Analysis of the diffraction symmetry revealed the Laue class $6/m$ as the highest possible symmetry with a reasonable R_{int} = 0.044. Systematic extinctions with 00ℓ reflections only observed for $\ell = 2n$ suggest the presence of a screw axis 6_3, and consequently the space group $P6_3/m$ (176) was chosen. Note that several 00ℓ reflections ($\ell = 2n+1$) violate the extinction rule, however, the intensity of those reflections is rather small (<7) compared to the highest intensity (~10000), and thus can be disregarded. Selected area electron diffraction (SAED) was performed to confirm the symmetry and unit cell parameters obtained from single crystal diffraction.

Structural solution employing program SIR92[1] was rather straightforward, revealing a similar set of atomic coordinates as in $Sm_{10}Ni_{20.8}P_{15}$. Structural refinement with program SHELXL-97[2] quickly converged to an R_F value of ~12 %. The refined composition at this stage (Ce22.5Ni47.2Si30.3, in at. %) deviates from the EPMA value (Ce24.6Ni43.5Si31.9, in at. %). However, at this point several sets of residual electron density close to each other were observed near the screw axis 6_3 suggesting the existence of severe disorder around 6_3, typical for compounds in this family. **Figure S3** shows the difference Fourier map at z = 0.25 around the origin. Following triangular arrangements of Ni and Si atoms in the lattice, it is reasonable to assign the peak labelled as Q1 as a partially filled Si site.

Moreover, distances around this electron density comply well with Ni-Si distances, ranging from 0.219 nm to 0.246 nm. Further refinement resulted in an occupancy value of ~0.6 Si in Q1. Since the overall composition lacks some cerium atoms, the second peak (Q2) located next to Q1 was assigned as a partially filled Ce site with occupancy of ~0.3. At this stage, the refined composition became closer to the EPMA composition, however, several small residual electron densities with magnitude ranging from 5 to 10×10^3 e^-/nm^3 near the 6_3-axis remained. Those residual densities were assigned as additional partially filled Ni and Si sites with small occupancies of less than 0.2.

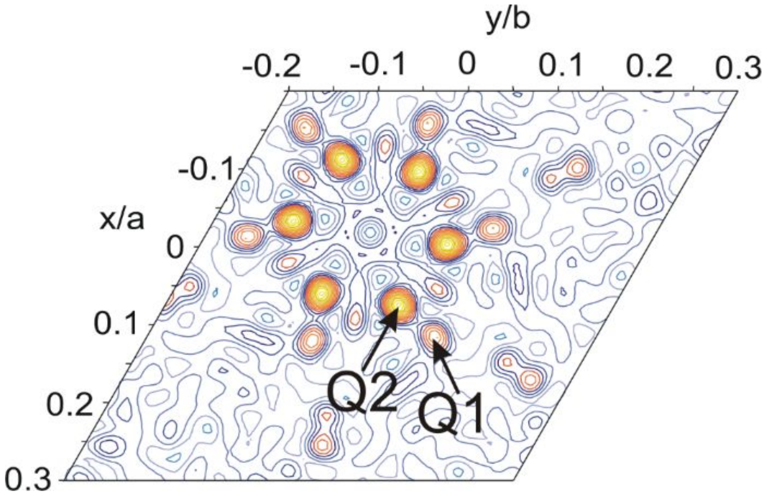

Figure S3. Difference Fourier map at z = 0.25, yielding several residual electron densities near the 6_3-screw axis at [0,0,z].

Table S1. Refined atomic position for $La_{20+x}Ni_{36+y}Si_{30-z}$ and $Ce_{20+x}Ni_{36+y}Si_{30-z}$ SC-1, $Ce_{20+x}Ni_{36+y}Si_{30-z}$ SC-2.

**Compound**	**$La_{20+x}Ni_{36+y}Si_{30-z}$**	**$Ce_{20+x}Ni_{36+y}Si_{30-z}$ SC-1**	**$Ce_{20+x}Ni_{36+y}Si_{30-z}$ SC-2**
RE1 in 6*h* (*x*, *y*, ¼); occ<br>U_{11};U_{22};U_{33};U_{23}=U_{13}=0;U_{12}	*x*=0.27858(3); *y*= 0.08123(3); 1 La<br>0.0156(3);0.0081(2);0.0088(2);0.0074(2)	*x*=0.28114(5); *y*= 0.08377(4); 1 Ce<br>0.0165(4);0.0069(3);0.0069(3);0.0067(3)	*x*=0.28019(5); *y*= 0.08314(4); 1 Ce<br>0.0155(4);0.0070(3);0.0068(4);0.0067(3)
RE2 in 6*h* (*x*, *y*, ¼); occ<br>U_{11};U_{22};U_{33};U_{23}=U_{13}=0;U_{12}	*x*=0.45464(3); *y*= 0.29496(3); 1 La<br>0.0090(2);0.0073(2);0.0074(2);0.0049(2)	*x*=0.45579(4); *y*= 0.29651(4); 1 Ce<br>0.0088(3);0.0070(3);0.0056(3);0.0047(3)	*x*=0.45564(4); *y*= 0.29637(4); 1 Ce<br>0.0088(3);0.0072(3);0.0056(3);0.0048(3)
RE3 in 6*h* (*x*, *y*, ¼); occ<br>U_{11};U_{22};U_{33};U_{23}=U_{13}=0;U_{12}	*x*=0.49345(3); *y*= 0.12175(3); 1 La<br>0.0072(2);0.0063(2);0.0072(2);0.0033(2)	*x*=0.49376(4); *y*= 0.12181(4); 1 Ce<br>0.0065(3);0.0057(3);0.0059(3);0.0028(2)	*x*=0.49366(4); *y*= 0.12179(4); 1 Ce<br>0.0069(3);0.0060(3);0.0060(3);0.0031(3)
RE4 in 2*d* (⅔, ⅓, ¼); occ<br>U_{11}=U_{22};U_{33};U_{23}=U_{13}=0;U_{12}	1 La<br>0.0084(2); 0.0089(4); 0.0042(2)	1 Ce<br>0.0084(3);0.0070(5);0.0028(2)	1 Ce<br>0.0087(4);0.0071(6);0.0043(2)
M1a in 12*i* (*x*, *y*, *z*); occ<br>U_{11};U_{22};U_{33};U_{23};U_{13};U_{12}	*x*=0.082(2); *y*= 0.0262(8); *z*= 0.135(4); 0.097(7) Ni<br>U_{iso}= 0.038(6)	*x*=0.0789(7); *y*= 0.0253(6); *z*= 0.138(3); 0.14(1) Ni<br>U_{iso}= 0.018(2)	*x*=0.081(1); *y*= 0.0262(8); *z*= 0.145(4); 0.11(1) Ni<br>U_{iso}= 0.018(3)
M1b in 6*h* (*x*, *y*, ¼); occ<br>U_{11}=U_{22};U_{33};U_{23}=U_{13}=0;U_{12}	*x*=0.111(1); *y*= 0.034(1); 0.028(5) 1 La<br>U_{iso}= 0.004(7)	*x*=0.1145(8); *y*= 0.0368(7); 0.19(1) Ni<br>U_{iso}= 0.018(2)	*x*=0.110(1); *y*= 0.0351(8); 0.16(1) Ni<br>U_{iso}= 0.018(3)
M1c in 6*h* (*x*, *y*, ¼); occ<br>U_{11}=U_{22};U_{33};U_{23}=U_{13}=0;U_{12}	*x*=0.035(1); *y*= 0.002(1); 0.094(8) 1 Ni<br>U_{iso}= 0.030(6)	*x*=0.051(2); *y*= 0.013(2); 0.08(1) Ni<br>U_{iso}= 0.018(2)	*x*=0.042(2); *y*= 0.007(2); 0.08(1) Ni<br>U_{iso}= 0.018(3)

M1d in 6*h* (*x*, *y*, ¼); occ<br>$U_{11}=U_{22};U_{33};U_{23}=U_{13}=0;U_{12}$	-	*x*=0.155(4); *y*= 0.057(4); 0.07(1) Si<br>U_{iso}= 0.018(2)	*x*=0.164(3); *y*= 0.061(3); 0.08(2) Si<br>U_{iso}= 0.018(3)
Ni2 in 6*h* (*x*, *y*, ¼); occ<br>$U_{11};U_{22};U_{33};U_{23}=U_{13}=0;U_{12}$	*x*=0.02675(7); *y*= 0.36807(7); 1<br>0.0070(5);0.0097(5);0.0097(6);0.0035(4)	*x*=0.0268(1); *y*= 0.36928(9); 1<br>0.0066(7);0.0084(7);0.0067(7);0.0035(6)	*x*=0.0268(1); *y*= 0.3692(1); 1<br>0.0073(8);0.0085(8);0.0065(8);0.0039(7)
Ni3a in 6*h* (*x*, *y*, ¼); occ<br>$U_{11};U_{22};U_{33};U_{23}=U_{13}=0;U_{12}$	*x*=0.0552(6); *y*= 0.1987(2); 0.46(3) 1<br>U_{iso}= 0.013(2)	*x*=0.0640(1); *y*= 0.2022(1); 0.96(1) Ni<br>0.027(1);0.018(1);0.024(1);0.011(1)	*x*=0.0636(2); *y*= 0.2022(1); 0.95(1) Ni<br>0.034(2);0.018(1);0.022(1);0.015(1)
Ni3b in 12*i* (*x*, *y*, *z*); occ<br>$U_{11};U_{22};U_{33};U_{23};U_{13};U_{12}$	*x*=0.0746(9); *y*= 0.2033(3); *z*= 0.293(2); 0.20(2) Ni<br>U_{iso}= 0.019(2)	-<br>-	-<br>-
Ni4 in 6*h* (*x*, *y*, ¼); occ<br>$U_{11};U_{22};U_{33};U_{23}=U_{13}=0;U_{12}$	*x*=0.20274(7); *y*= 0.58183(7); 1<br>0.0092(5);0.0090(5);0.0128(6);0.0102(2)	*x*=0.2039(1); *y*= 0.5831(1); 1<br>0.0081(7);0.0076(7);0.0170(9);0.0039(6)	*x*=0.2039(1); *y*= 0.5830(1); 1<br>0.0094(8);0.0076(8);0.0169(9);0.0047(7)
Ni5 in 6*h* (*x*, *y*, ¼); occ<br>$U_{11};U_{22};U_{33};U_{23}=U_{13}=0;U_{12}$	*x*=0.24064(7); *y*= 0.40493(7); 1<br>0.0109(5);0.0106(6);0.0139(6);0.0049(5)	*x*=0.2418(1); *y*= 0.4085(1); 1<br>0.0093(8);0.0084(8);0.0186(9);0.0054(6)	*x*=0.2416(1); *y*= 0.4080(1); 1<br>0.0096(8);0.0093(8);0.018(1);0.0058(7)
Ni6 in 6*h* (*x*, *y*, ¼); occ<br>$U_{11};U_{22};U_{33};U_{23}=U_{13}=0;U_{12}$	*x*=0.28468(8); *y*= 0.23526(8); 1<br>0.0191(6);0.0172(6);0.0089(6);0.0150(5)	*x*=0.2838(1); *y*= 0.2361(1); 1<br>0.0136(8);0.0114(8);0.0075(8);0.0098(7)	*x*=0.2835(1); *y*= 0.2357(1); 1<br>0.0139(9);0.0120(8);0.0072(8);0.0101(7)
Ni7 in 6*h* (*x*, *y*, ¼); occ<br>$U_{11};U_{22};U_{33};U_{23}=U_{13}=0;U_{12}$	*x*=0.55420(7); *y*= 0.01077(7); 1<br>0.0123(6);0.0076(5);0.0111(6);0.0046(4)	*x*=0.5534(1); *y*= 0.00995(9); 1<br>0.0095(8);0.0064(7);0.0079(8);0.0034(6)	*x*=0.5533(1); *y*= 0.0100(1); 1<br>0.0094(8);0.0070(8);0.0081(8);0.0035(7)
Si1 in 6*h* (*x*, *y*, ¼); occ<br>$U_{11};U_{22};U_{33};U_{23}=U_{13}=0;U_{12}$	*x*=0.0721(1); *y*= 0.4973(1); 1<br>0.008(1);0.007(2);0.007(2);0.0047(9)	*x*=0.0752(2); *y*= 0.4995(2); 1<br>0.005(1);0.006(1);0.008(1);0.003(1)	*x*=0.0754(2); *y*= 0.4997(2); 1<br>0.005(2);0.008(2);0.006(2);0.005(1)
Si2 in 6*h* (*x*, *y*, ¼); occ<br>$U_{11};U_{22};U_{33};U_{23}=U_{13}=0;U_{12}$	*x*=0.1107(2); *y*= 0.3235(2); 1<br>0.012(1);0.017(1);0.008(1);0.010(1)	*x*=0.1131(2); *y*= 0.3280(2); 1<br>0.006(1);0.011(2);0.007(2);0.006(1)	*x*=0.1127(2); *y*= 0.3277(2); 1<br>0.006(2);0.010(2);0.007(2);0.006(1)
M3a in 6*h* (*x*, *y*, ¼); occ<br>$U_{11};U_{22};U_{33};U_{23}=U_{13}=0;U_{12}$	*x*=0.149(1); *y*= 0.148(1); 0.37(4) Si<br>U_{iso}= 0.024(4)	*x*=0.1510(4); *y*= 0.1548(5); 0.63(2) Si<br>0.019(4);0.034(5);0.012(3);0.016(3)	*x*=0.1484(5); *y*= 0.1503(6); 0.66(3) Si<br>0.031(5);0.053(7);0.016(4);0.024(5)
M3b in 6*h* (*x*, *y*, ¼); occ<br>$U_{11};U_{22};U_{33};U_{23}=U_{13}=0;U_{12}$	*x*=0.139(2); *y*= 0.121(2); 0.33(6) Si<br>U_{iso}= 0.025(6)	*x*=0.1054(2); *y*= 0.0884(2); 0.31(1) Ce<br>0.034(2);0.031(2);0.042(2);0.028(2)	*x*=0.1047(2); *y*= 0.0889(2); 0.36(1) Ce<br>0.043(2);0.042(2);0.041(2);0.037(2)
M3c in 6*h* (*x*, *y*, ¼); occ<br>$U_{11};U_{22};U_{33};U_{23}=U_{13}=0;U_{12}$	*x*=0.0979(6); *y*= 0.0823(5); 0.14(1) La<br>U_{iso}= 0.006(2)	-	-
M3d in 12*i* (*x*, *y*, z); occ<br>$U_{11};U_{22};U_{33};U_{23};U_{13};U_{12}$	*x*=0.1158(6); *y*=0.0965(5); *z*= 0.312(2); 0.12(1) La<br>U_{iso}= 0.014(2)	-	-
Si4 in 6*h* (*x*, *y*, ¼); occ<br>$U_{11};U_{22};U_{33};U_{23}=U_{13}=0;U_{12}$	*x*=0.2877(1); *y*= 0.5365(2); 1<br>0.005(1);0.010(1);0.012(1);0.0046(9)	*x*=0.2881(2); *y*= 0.5377(2); 1<br>0.009(2);0.011(2);0.012(2);0.007(1)	*x*=0.2884(2); *y*= 0.5375(2); 1<br>0.008(2);0.010(2);0.014(2);0.004(1)
Si5 in 6*h* (*x*, *y*, ¼); occ	*x*=0.3281(1); *y*= 0.3633(2); 1	*x*=0.3271(2); *y*= 0.3654(2); 1	*x*=0.3269(2); *y*= 0.3651(2); 1

U_{11};U_{22};U_{33};U_{23}=U_{13}=0;U_{12}	0.009(2);0.010(1);0.009(1);0.0045(9)	0.008(2);0.011(2);0.008(2);0.004(1)	0.007(2);0.010(2);0.007(2);0.004(1)
Residual electron density; max; min in [electrons/nm^3] × 1000	2.46; -2.72	2.32; -4.85	2.54; -3.04

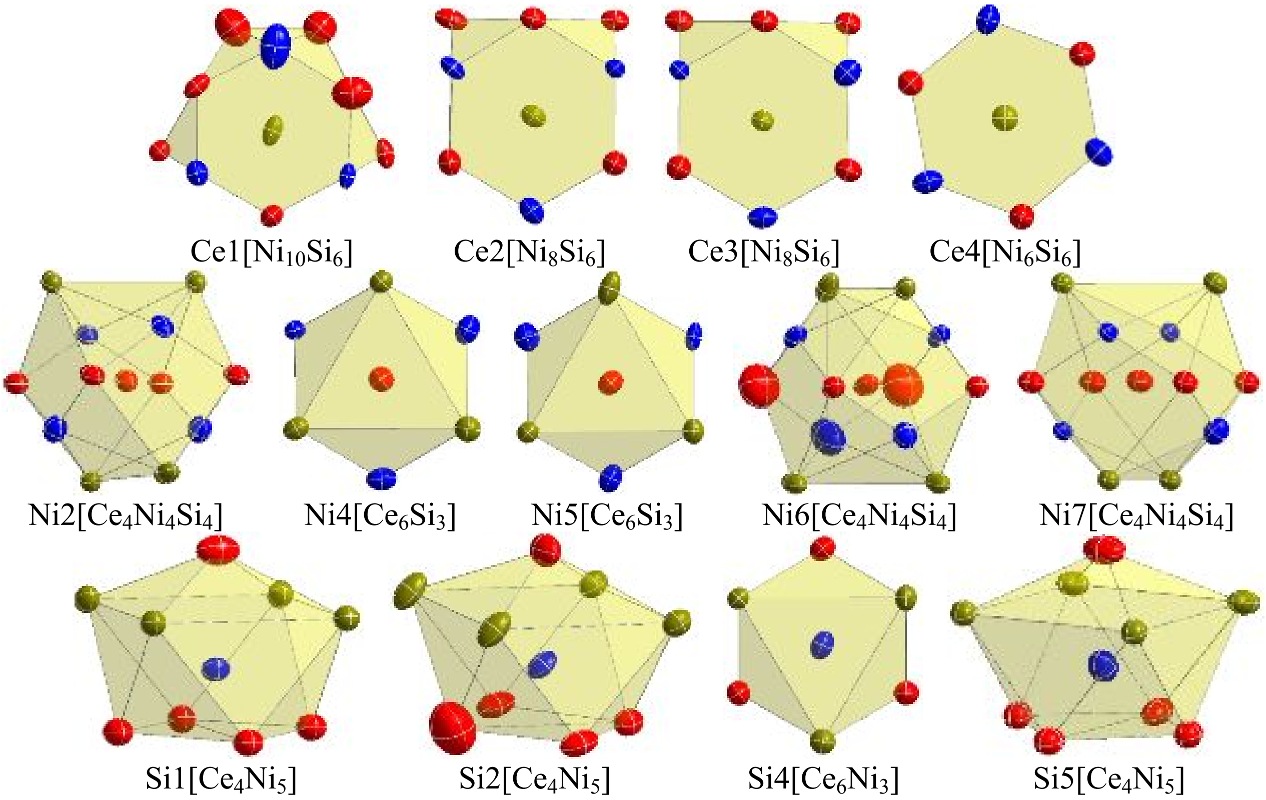

Figure S4. Coordination polyhedra of atoms in τ-$Ce_{20+x}Ni_{36+y}Si_{30-z}$ outside the disordered area. Atoms are displayed with their anisotropic thermal displacement ellipsoids as derived from X-ray single crystal refinement at 90% probability level (see **Table S1**). Ce-atoms in green, Ni-atoms in red and Si-atoms in blue colour.

Crystal structure of τ'-$Ce_{30+x}Ni_{50+y}Si_{42-z}$ – a new member of the homologue series $RE_{n(n+1)+x}Ni_{n(n+5)+y}Si_{(n+1)(n+2)-z}$ (n = 5)

The set of atom positions of $Tb_{15}Ni_{28}P_{21}$ was used as input for the Rietveld refinement, and the refinement quickly progressed to a reasonable R_I-value of ~7 %. At this stage, however, the refined composition ($Ce_{23.4}Ni_{43.8}Si_{32.8}$ in at. %) still deviates from the EPMA composition $Ce_{27.0}Ni_{41.3}Si_{31.7}$ in at. %, indicating that some Ce atoms are missing from the structure. A difference Fourier map in **Figure S5** shows some small residual electron densities around the 6-fold axis, which were assigned as Ce.

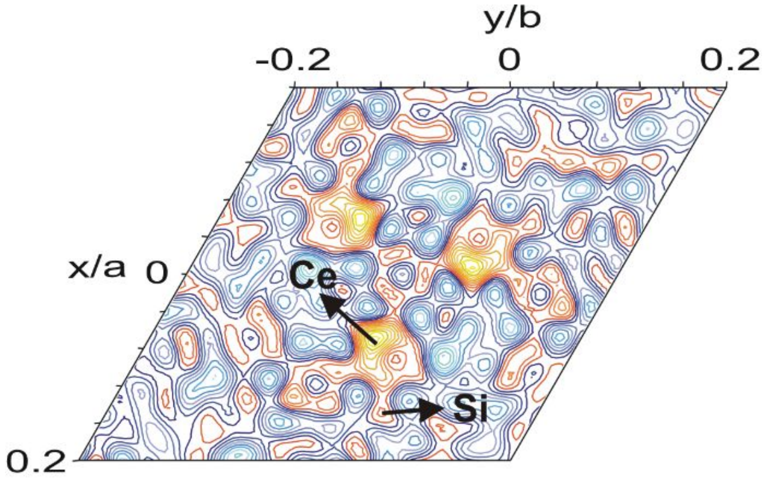

Figure S5. Difference Fourier map at z = 0.25 for alloy $Ce_{27.0}Ni_{41.3}Si_{31.7}$, showing some residual density around the 6_3-screw axis.

Table S2. Refined atomic position of $Ce_{30+x}Ni_{50+y}Si_{42-z}$. The atomic positions are standardized according to $Tb_{15}Ni_{28}P_{21}$.

Compound	$Ce_{30+x}Ni_{50+y}Si_{42-z}$
Ce1 in 6*h* (*x*, *y*, ¼); occ; B_{iso}	*x*= 0.2314(1); *y*= 0.1706(1); 1; 0.49(1)
Ce2 in 6*h* (*x*, *y*, ¼); occ; B_{iso}	*x*= 0.4112(1); *y*= 0.3202(1); 1; 0.46(1)
Ce3 in 6*h* (*x*, *y*, ¼); occ; B_{iso}	*x*= 0.3828(1); *y*= 0.1416(1); 1; 0.55(1)
Ce4 in 6*h* (*x*, *y*, ¼); occ; B_{iso}	*x*= 0.5596(1); *y*= 0.2933(1); 1;0.54(1)
Ce5 in 6*h* (*x*, *y*, ¼); occ; B_{iso}	*x*= 0.5312(1); *y*= 0.1158(1); 1; 0.37(1)
M6a in 6*h* (*x*, *y*, ¼); occ; B_{iso}	*x*= 0.0949(3); *y*= 0.0137(2); 0.37(1) Ce; 0.83(1)
M6b in 6*h* (*x*, *y*, ¼); occ; B_{iso}	*x*= 0.0057(9); *y*= 0.1407(7); 0.56(1) Si; 0.83(1)
Ni1 in 2*d* (⅓, ⅔, ¼); occ; B_{iso}	1; 0.24(1)
Ni3 in 6*h* (*x*, *y*, ¼); occ; B_{iso}	*x*= 0.1146(3); *y*= 0.1710(3); 0.90(1); 0.20(1)
Ni4 in 6*h* (*x*, *y*, ¼); occ; B_{iso}	*x*= 0.2424(3); *y*= 0.0440(3); 0.94(1); 0.81(1)
Ni5 in 6*h* (*x*, *y*, ¼); occ; B_{iso}	*x*= 0.2828(3); *y*= 0.3192(3); 1; 0.54(1)
Ni6 in 6*h* (*x*, *y*, ¼); occ; B_{iso}	*x*= 0.3883(3); *y*= 0.0169(3); 1; 0.49(1)
Ni7 in 6*h* (*x*, *y*, ¼); occ; B_{iso}	*x*= 0.0064(3); *y*= 0.5397(3); 1;0.42(1)
Ni8 in 6*h* (*x*, *y*, ¼); occ; B_{iso}	*x*= 0.3085(3); *y*= 0.4860(3); 0.94(1); 0.59
Ni9 in 6*h* (*x*, *y*, ¼); occ; B_{iso}	*x*= 0.1525(3); *y*= 0.5175(3); 1; 0.52(1)
Ni10 in 6*h* (*x*, *y*, ¼); occ; B_{iso}	*x*= 0.1275(3); *y*= 0.3339(3); 0.98(1); 0.58
Si1 in 6*h* (*x*, *y*, ¼); occ; B_{iso}	*x*= 0.1784(5); *y*= 0.2769(5); 1; 0.54(1)
Si2 in 6*h* (*x*, *y*, ¼); occ; B_{iso}	*x*= 0.3469(5); *y*= 0.4188(6); 1; 0.54(1)
Si3 in 6*h* (*x*, *y*, ¼); occ; B_{iso}	*x*= 0.0492(6); *y*= 0.4783(5); 1; 0.54(1)
Si4 in 6*h* (*x*, *y*, ¼); occ; B_{iso}	*x*= 0.2268(7); *y*= 0.6247(7); 1; 0.54(1)
Si5 in 6*h* (*x*, *y*, ¼); occ; B_{iso}	*x*= 0.0176(5); *y*= 0.2955(5); 1; 0.54(1)
Si6 in 6*h* (*x*, *y*, ¼); occ; B_{iso}	*x*= 0.2025(6); *y*= 0.4462(6); 1; 0.54(1)

Crystal structure of monoclinic $Ce_3Ni_4Si_2$ (formerly $CeNi_{1.3}Si_{0.7}$) – a new homologue series

Analysis of systematic extinctions suggested two possible space groups *Cc* (No. 9) and *C*2/*c* (No. 15). Despite the E-test giving a hint for centrosymmetry, structure solution employing direct methods was performed first in the non-centrosymmetric space group *Cc*, and quickly converged to an R_F value of ~4 %. Analysis of missing symmetry with program PLATON[3] suggested the centrosymmetric space group of *C*2/*c*, and the structure was furthermore refined in this space group.

Table S3. Refined atomic position for $Ce_3Ni_4Si_2$ and $La_3Ni_4Si_2$.

**Compound**	**$Ce_3Ni_4Si_2$**	**$La_3Ni_4Si_2$**
RE1 in 8*f* (*x*, *y*, *z*); occ	*x*= 0.15522(2); *y*= 0.11123(2); *z*= 0.45281(2); 1.00(1) Ce	*x*= 0.1562(1); *y*= 0.1012(5); *z*= 0.4566(3); 1 La
U_{11};U_{22};U_{33};U_{23};U_{13};U_{12}	0.0091(1);0.0082(1);0.0098(1);0.0005(1);0.0028(1);0.0004(1)	B_{iso} = 0.68(1)
RE2 in 4*e* (0, *y*,¼); occ	*y*= 0.62405(3); 1.00(1) Ce	*y*= 0.6259(7); 1 La
U_{11};U_{22};U_{33};U_{23}=U_{12}=0;U_{13}	0.0071(1);0.0091(1);0.0090(1);0.0017(1)	B_{iso} = 0.32(1)
Ni1 in 8*f* (*x*, *y*, *z*); occ	*x*= 0.03596(2); *y*= 0.14764(6); *z*= 0.11324(5); 1.00(1)	*x*= 0.0395(3); *y*= 0.145(1); *z*= 0.1025(6); 1
U_{11};U_{22};U_{33};U_{23};U_{13};U_{12}	0.0094(1);0.0122(2);0.0122(2);-0.0014(1);0.0028(1);-0.0010(1)	B_{iso} = 0.20(1)
Ni2 in 8*f* (*x*, *y*, *z*); occ	*x*= 0.28790(2); *y*= 0.15412(6); *z*= 0.16170(4); 1.00(1)	*x*= 0.2895(4); *y*= 0.142(1); *z*= 0.1510(7); 0.98(1)
U_{11};U_{22};U_{33};U_{23};U_{13};U_{12}	0.0092(1);0.0142(2);00085(1);0.0019(1);0.0017(1);0.0022(1)	B_{iso} = 0.91 (1)
Si1 in 8*f* (*x*, *y*, *z*); occ	*x*= 0.16069(5); *y*= 0.3912(1); *z*= 0.1102(1) ; 1.00(1)	*x*= 0.1624(7); *y*= 0.395(1); *z*= 0.120(1); 1
U_{11};U_{22};U_{33};U_{23};U_{13};U_{12}	0.0088(3);0.0086(3);00091(3);-0.0005(2);0.0033(2);-0.007(2)	B_{iso} = 0.27(1)

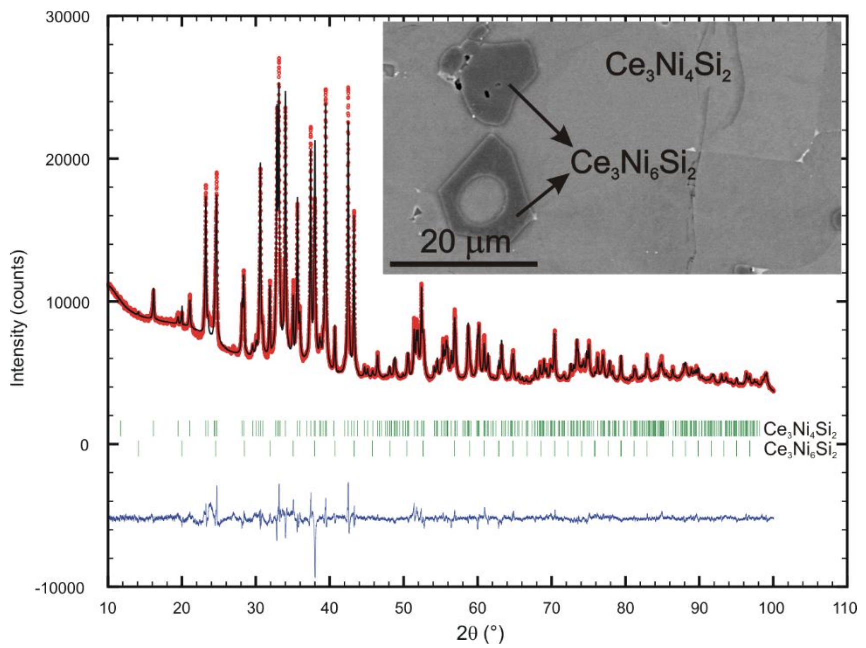

Figure S6. Rietveld refinement of sample with overall composition Ce32.6Ni45.0Si22.4 (in at. %).

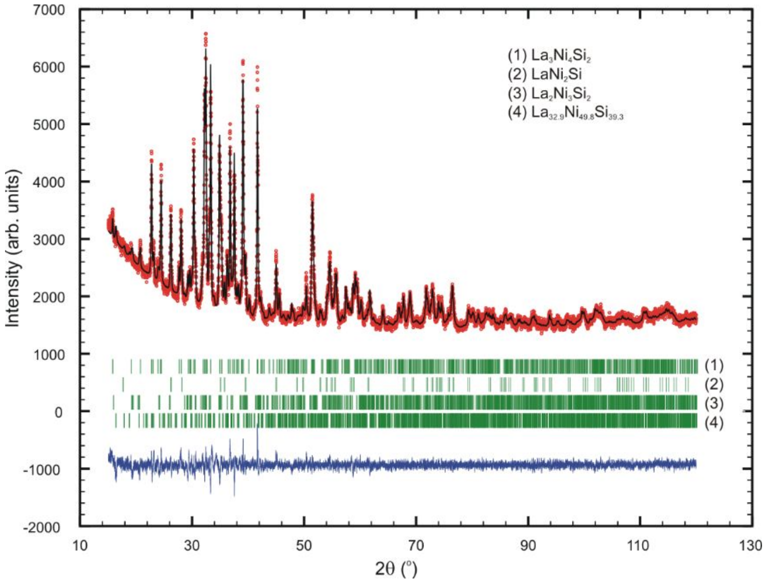

Figure S7. Rietveld refinement of sample with overall composition La33.3Ni44.4Si22.3 (in at. %).

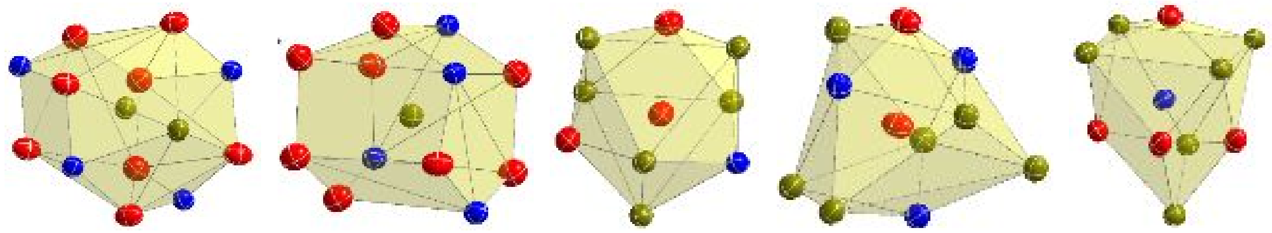

Ce1[$CeNi_8Si_4$] Ce2[Ni_8Si_4] Ni1[Ce_6Ni_2Si] Ni2[Ce_6NiSi_3] Si[Ce_6Ni_4]

Figure S8. Coordination polyhedra for all atoms in $Ce_3Ni_4Si_2$. Atoms are displayed with their anisotropic thermal displacement ellipsoids as derived from X-ray single crystal refinement at 90% probability level (see **Table S3**). Ce-atoms in green, Ni-atoms in red and Si-atoms in blue colour.

References

(1) Altomare, A.; Cascarano, G.; Giacovazzo, C.; Guagliardi, A.; Burla, M. C.; Polidori, G.; Camalli, M. SIR92 – a Program for Automatic Solution of Crystal Structures by Direct Methods. *J. Appl. Crystallogr.* **1994**, *27* (3), 435–435. https://doi.org/10.1107/S002188989400021X.

(2) Sheldrick, G. M. Crystal Structure Refinement with SHELXL. *Acta Crystallogr. Sect. C Struct. Chem.* **2015**, *71* (1), 3–8. https://doi.org/10.1107/S2053229614024218.

(3) Spek, A. L. Single-Crystal Structure Validation with the Program PLATON. *J. Appl. Crystallogr.* **2003**, *36* (1), 7–13. https://doi.org/10.1107/S0021889802022112.
